# Fine Mapping of the *Co-12* Anthracnose Resistance Gene in the Andean Common Bean Cultivar in Brazil

**DOI:** 10.3390/plants15060931

**Published:** 2026-03-18

**Authors:** Jaqueline Bezerra da Silva, Maria Celeste Gonçalves-Vidigal, Pedro Soares Vidigal Filho, Giselly Figueiredo Lacanallo, Mariana Vaz Bisneta, Giseli Valentini, Larissa Fernanda Sega Xavier

**Affiliations:** 1 Programa de Pós-Graduação em Genética e Melhoramento, Universidade Estadual de Maringá, Maringá 87020900, PR, Brazil; jaquelinebezerra.agro@gmail.com (J.B.d.S.); vidigalfilhop@gmail.com (P.S.V.F.); gflacanallo@uem.br (G.F.L.); larissafsx@gmail.com (L.F.S.X.); 2 Department of Plant Science and Plant Pathology, Montana State University, Bozeman, MT 59717, USA; giseli.valentini@montana.edu

**Keywords:** *Colletotrichum lindemuthianum*, *Phaseolus vulgaris*, genetic resistance

## Abstract

The common bean (*Phaseolus vulgaris* L.) cultivar Jalo Vermelho carries the *Co-12* gene, which confers resistance to both Andean and Mesoamerican races of *Colletotrichum lindemuthianum*. Despite its importance for breeding programs, the genomic location and candidate genes underlying this resistance remain poorly defined. The *Co-12* locus was fine-mapped using a biparental population derived from the cross Jalo Vermelho × Crioulo 159. A total of 172 F_2_ plants were used to generate 172 F_2:3_ families, which were phenotyped after inoculation with race 1545 of *C. lindemuthianum*. Segregation analysis confirmed a 1:2:1 Mendelian ratio, consistent with a single dominant resistance gene. Genotyping of resistant and susceptible plants using the BARBean6K_3 Illumina BeadChip (5398 SNP markers) mapped *Co-12* to chromosome Pv04, between 1695 bp (ss715649768) and 9,651,954 bp (ss715646644). Subsequent fine mapping with simple sequence repeat (SSR) markers delimited the locus to a 41 kb genomic interval flanked by BARCPVSSR04557 and BARCPVSSR04570. Within this region, three candidate genes were identified, including one encoding a gamma-glutamyl-GABA enzyme and two encoding lipid transfer proteins (LTP2). Lipid transfer proteins are widely recognized components of plant defense; however, their association with anthracnose resistance in the common bean has not been previously reported. The identification of LTP2 genes within the *Co-12* interval suggests a previously unrecognized resistance mechanism and expands the current understanding of host defense pathways in *Phaseolus vulgaris*. The markers identified here provide valuable tools for marker-assisted selection and will facilitate efficient introgression of *Co-12* into common bean cultivars.

## 1. Introduction

The genus *Phaseolus* comprises approximately 76 species, of which five have been domesticated: *P. vulgaris* (common bean), *P. lunatus* (lima bean), *P. coccineus* (ayocote bean), *P. acutifolius* (tepari bean), and *P. dumosus* [1,2]. Among these, the common bean (*P. vulgaris* L.) is the most widely cultivated and economically important species, serving as a major source of proteins, micronutrients, carbohydrates, dietary fiber, antioxidant molecules, phenolic compounds, and unsaturated fatty acids. Consequently, it constitutes a fundamental component of human nutrition for millions of people worldwide [3,4,5]. India, Myanmar, and Brazil are currently the leading producers of the common bean. In Brazil, total grain production for the 2025/2026 harvest is estimated at approximately 3.0 million tons [6], underscoring the strategic importance of the common bean for national food security and agribusiness.

Despite its agronomic importance, common bean production is constrained by several abiotic and biotic stresses. Among biotic stresses, anthracnose—caused by the fungus *Colletotrichum lindemuthianum* (Saccardo and Magnus) Briosi and Cavara—stands out as one of the most destructive diseases affecting this crop. Seeds represent the primary source of inoculum and pathogen dissemination, and disease damage is most severe during the early stages of plant development. Consequently, anthracnose can cause severe reductions in yield and grain quality [7,8,9].

To date, approximately 298 physiological races of *C. lindemuthianum* have been reported worldwide [10]. This high level of genetic and pathogenic variability greatly complicates disease management, requiring integrated strategies that include the use of certified seeds, chemical control, crop rotation, and, most importantly, resistant cultivars. Among these measures, the deployment of resistant cultivars is considered the most efficient, economical, and environmentally sustainable approach for anthracnose control [11,12]. Accordingly, common bean breeding programs play a central role in identifying resistance sources to diverse pathogen races and in developing cultivars with broad and durable resistance through gene pyramiding strategies [13].

*C. lindemuthianum* is traditionally classified into two major groups based on its virulence patterns: Mesoamerican and Andean. This classification reflects the genetic structure of the common bean, which is composed of two primary gene pools, Middle American and Andean. Andean races of the pathogen predominantly infect Andean bean genotypes, whereas Mesoamerican races exhibit a broader virulence spectrum, infecting both gene pools, with preferential virulence toward Middle American genotypes [14].

The anthracnose resistance genes are denoted by the symbol “Co” followed by a number or letter [13,15]. Several genes and allelic series have been described in both the Andean and Middle American gene pools of the common bean [16]. A total of 11 dominant genes and allelic series have been described in the Middle American gene pool: *Co-2* (and allelic series *Co-2*^A252^ and *Co-2*^AB136^) [17,18,19,20]; *Co-3* (and allelic series *Co-3*^2^, *Co-3*^3^, *Co-3*^4^, *Co-3*^5^, and *Co-3*^A252^) [19,21,22,23,24]; *Co-4* (and allelic series *Co-4*^2^ and *Co-4*^3^) [25,26,27,28,29,30,31,32]; *Co-5* (and allelic series *Co-5*^2^) [33,34,35]; *Co-6* [16,36]; *Co-11* [16]; *Co-16* [37]; *Co-17* [38]; *Co-u* [39]; *Co-v* [40]; and *CoPv09cC* [41].

In the Andean gene pool, 14 dominant genes and allelic series have been described: *Co-1* (and allelic series *Co-1*^2^, *Co-1*^3^, *Co-1*^4^, *Co-1*^5^, *Co-1*^HY^, and *Co-1*^x^) [16,23,42,43]; *CoPv01*^CDRK^ [16]; and allelic series *Co-3*^K^ [44], *Co-3*^M^ [20,33], *Co-3*^W^ [21], *Co-3*^cX^ [41], *Co-12* [45], *Co-13* [16], *Co-14* [16], *Co-15* [46], *Co-w* [39], *Co-x* [47], *Co-y* [39,48], *Co-z* [39,48], *Co-AC* [16], *Co-Pa* [49], *CoPv02cX* [41], and *Co-Bf* [50,51].

Seeds of the common bean cultivar Jalo Vermelho were originally collected from small farms in the state of Paraná, southern Brazil, in 2001 [52]. This Andean cultivar represents an important source of resistance, exhibiting effective defense against thirteen races of *C. lindemuthianum*, including races 9, 23, 31, 55, 64, 65, 81, 83, 89, 95, 449, 453, and 1545 [45,49,52]. Genetic characterization of the *Co-12* gene through inheritance and allelism tests demonstrated that Jalo Vermelho has a single dominant resistance gene located at a distinct locus, clearly different from previously described resistance genes [45]. Considering the relevance of this resistance source for common bean breeding programs, this study aimed to fine-map the *Co-12* gene in Jalo Vermelho and to identify closely linked molecular markers to support marker-assisted selection for anthracnose resistance.

## 2. Results

### 2.1. Inheritance of Resistance to Race 1545 of Colletotrichum lindemuthianum

**Figure 1:**
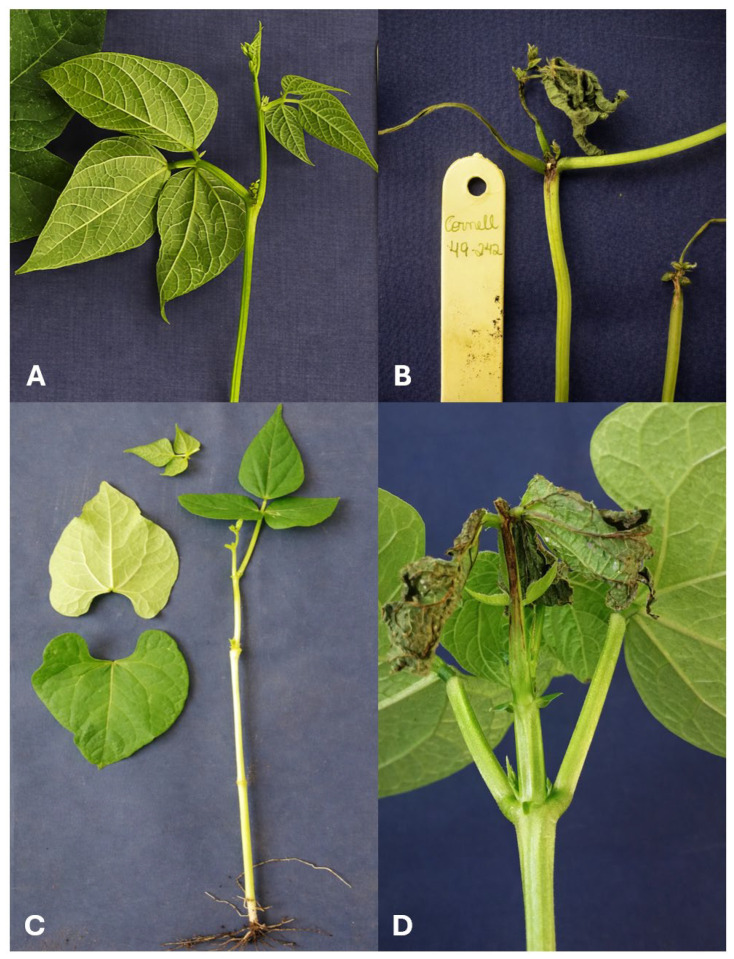
Phenotypic response to *Colletotrichum lindemuthianum* race 1545. (**A**) Widusa, the resistant control cultivar; (**B**) Cornell 49-242, the susceptible control cultivar; (**C**) Jalo Vermelho, the resistant parent; and (**D**) Crioulo 159, the susceptible parent.

**Figure 2:**
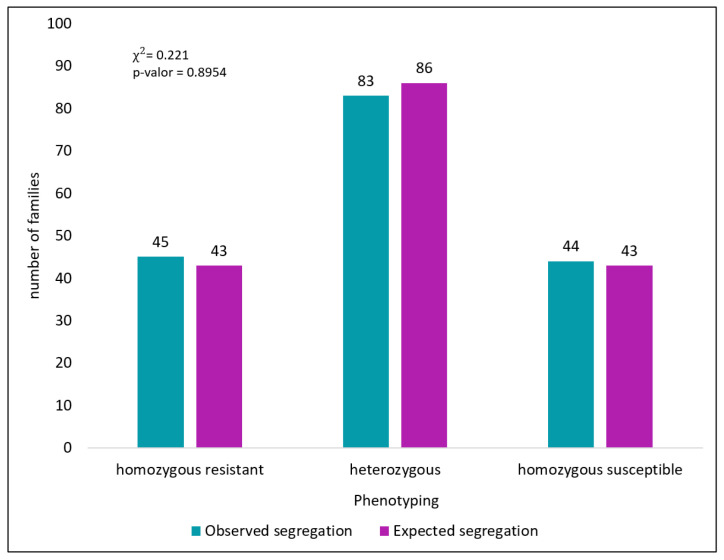
Representation of the chi-square analysis of 172 F_2:3_ families from the Jalo Vermelho × Crioulo 159 cross.

The Andean cultivar Jalo Vermelho showed complete resistance to race 1545 of *Colletotrichum lindemuthianum*, whereas the Mesoamerican cultivar Crioulo 159 was highly susceptible (Figure 1). The resistant control Widusa exhibited no disease symptoms, whereas the susceptible control Cornell 49-242 displayed typical anthracnose lesions, confirming the reliability of the inoculation procedure. Segregation analysis of the 172 F_2:3_ families derived from the Jalo Vermelho × Crioulo 159 cross fitted the expected Mendelian ratio of 1RR:2RS:1SS (χ^2^ = 0.221; *p* = 0.895) (Table 1; Figure 2), demonstrating that resistance to race 1545 in Jalo Vermelho is governed by a single dominant gene.

### 2.2. Mapping Using SNP Markers

**Figure 3:**
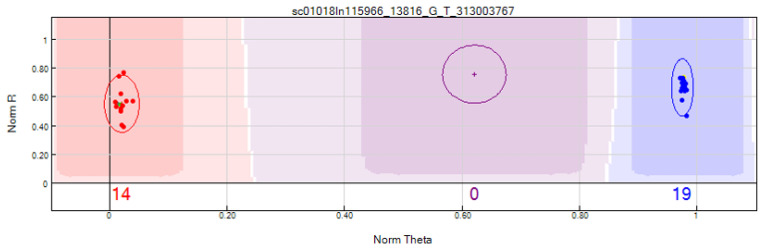
Representation of the BARCPV_1.0_Chr04_101676_G_T (ss715649770) SNP marker in 35 F_2_ plants from the Jalo Vermelho × Crioulo 159 cross. Green dot: Jalo Vermelho; Red dots: resistant plants; Yellow dot (It doesn’t appear in the image, but it’s in the middle of the blue dots): Crioulo 159; Blue dots: susceptible plants. Red circle: Resistant plants; Blue circle: susceptible plants; Purple circle: heterozygous plants (no heterozygous plants identified).

Genotyping of 17 resistant (Table S1) and 18 susceptible (Table S2) F_2_ plants with the BeadChip platform revealed 150 SNP markers associated with resistance to race 1545 (Table S3). These SNPs delimited the *Co-12* gene to a region spanning from 1695 bp (ss715649768) to 9,651,954 bp (ss715646644) on chromosome Pv04. Clear clustering of resistant and susceptible genotypes relative to the parental lines confirmed linkage between these markers and the resistance locus (Figure 3).

### 2.3. Mapping Using SSR Markers and a Linkage Map

A total of 22 SSR markers were developed for the region delimited by the SNP markers ss715649768 and ss715646644. All SSR markers were analyzed in the parental lines Jalo Vermelho and Crioulo 159, and the results revealed five polymorphic markers between the parents (Table 2). These markers were subsequently analyzed in the 172 F_2:3_ families (Table S4). The chromosomal region containing these five SSR markers was delimited by the SNP markers ss715649772 (33,578 bp) and ss715646246 (2,428,047 bp) based on the G19833 reference genome (v2.1), available at Phytozome: “https://phytozome-next.jgi.doe.gov/ (accessed on 11 December 2025)”.

**Figure 4:**
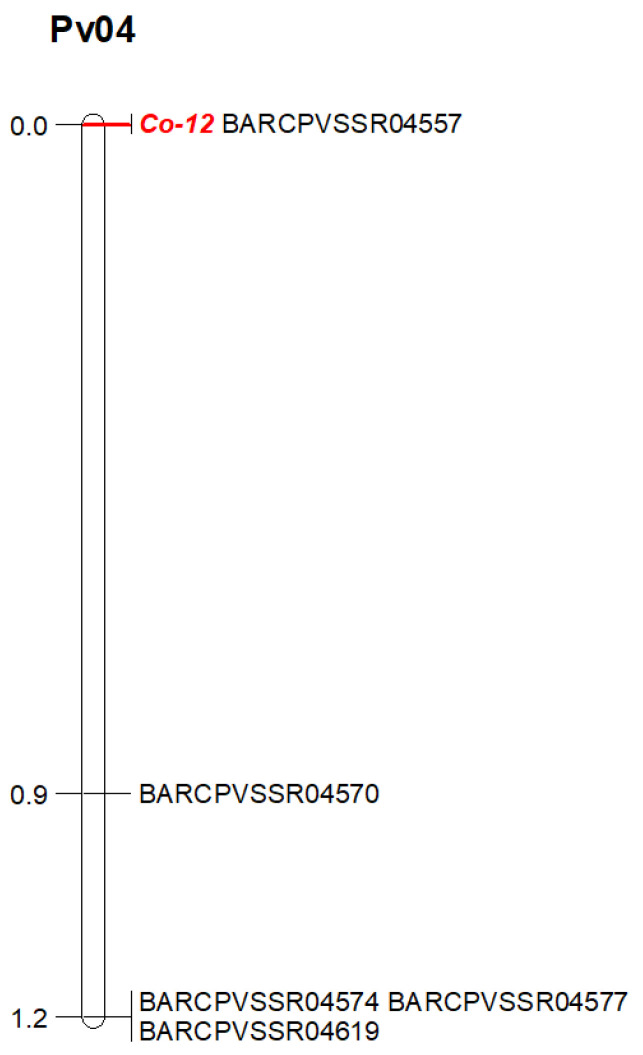
Genetic map of the anthracnose resistance gene in Jalo Vermelho (*Co-12*) on chromosome Pv04 constructed using 172 F_2_ plants from the Jalo Vermelho × Crioulo 159 cross, inoculated with race 1545 of *Colletotrichum lindemuthianum* and genotyped with five SSR markers. The distances between SSR markers and the *Co-12* gene are expressed in centimorgans (cM).

**Figure 5:**
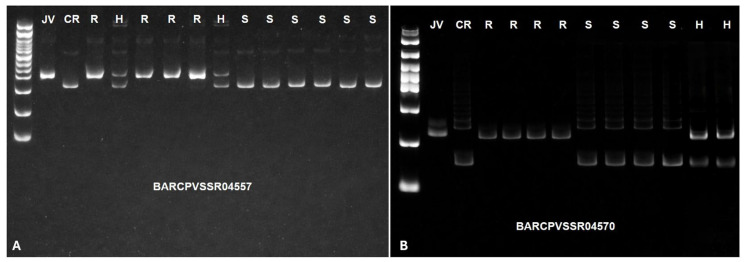
(**A**) PCR amplification profiles of the SSR markers BARCPVSSR04557 (**A**) and BARCPVSSR04570 (**B**), showing banding patterns of the resistant parental Jalo Vermelho (JV), the susceptible parental Crioulo 159 (CR), and the homozygous resistant (R), homozygous susceptible (S), and heterozygous (H) genotypes.

**Figure 6:**
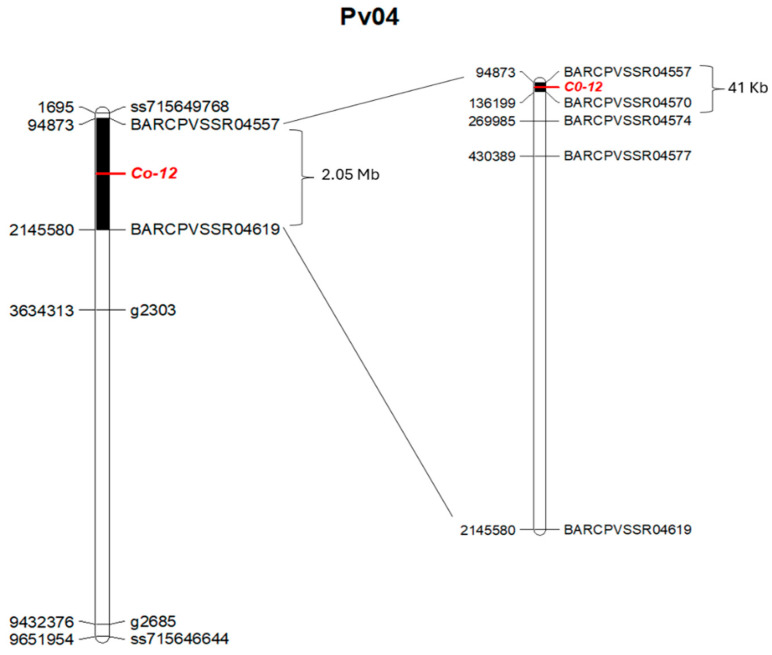
Physical mapping of the *Co-12* gene on chromosome Pv04 of the common bean. Map constructed using 35 F_2_ plants from the Jalo Vermelho × Crioulo 159 cross and genotyped with SNP markers (**left**). Physical map constructed using 172 F_2_ plants from the same cross and genotyped with SSR markers (**right**). SNP markers are represented by their base pair positions, whereas SSR markers are represented by the base pair positions corresponding to the start of their primers. All positions are based on genome version v2.1 (available at Phytozome). The markers g2303 and g2685 were linked to the genes in the Ouro Negro and Corinthiano cultivars, respectively [24,46].

The anthracnose resistance gene in Jalo Vermelho was closely linked to the marker BARCPVSSR04557 at 0.0 cM and located 0.9 cM from the marker BARCPVSSR04570 (Figure 4). The SSR banding patterns corresponding to homozygous resistant, homozygous susceptible, and heterozygous individuals are shown in the genotyping gels using the markers BARCPVSSR04557 and BARCPVSSR04570 (Figure 5). Based on the physical position of the SSR markers and the anthracnose reaction of the F_2:3_ families, four F_2_ plants exhibited recombination breakpoints between markers BARCPVSSR04557 (95 kb) and BARCPVSSR04570 (136 kb). These results delimit the anthracnose resistance gene in Jalo Vermelho to a 41 kb region on chromosome Pv04 (Figure 6; Table 3).

### 2.4. Candidate Genes and Associated Functions

**Figure 7:**
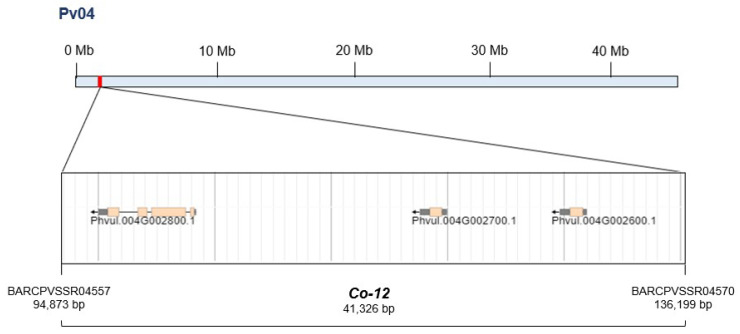
Candidate genes on chromosome Pv04 are located between the SSR markers BARCPVSSR04557 (94,873 bp) and BARCPVSSR04570 (136,199 bp), with arrows indicating gene orientation.

The 41 kb genomic region harboring the *Co-12* resistance locus, delimited by the markers BARCPVSSR04557 and BARCPVSSR04570 on chromosome Pv04, contains three annotated candidate genes in the common bean reference genome (Figure 7). These genes include Phvul.004G002800, which encodes a gamma-glutamyl-GABA protein, and Phvul.004G002700 and Phvul.004G002600, both of which encode lipid transfer proteins of the LTP2 family. The gene Phvul.004G002800 shows 71.9% sequence similarity to the *Arabidopsis thaliana* gene AT5G38200, which is associated with amino acid metabolism and cellular stress responses. The genes Phvul.004G002700.1 and Phvul.004G002600.1 exhibit 44.7% and 53.8% sequence similarity, respectively, with the *A. thaliana* gene AT3G18280.1, which encodes a lipid transfer protein involved in membrane stability, lipid transport, and defense-related processes (Table 4).

## 3. Discussion

Cultivars resistant to multiple races of *Colletotrichum lindemuthianum* are essential for breeding programs targeting durable anthracnose resistance. In this context, the Andean cultivar Jalo Vermelho represents a particularly valuable resistance source, as it is resistant to both Andean and Mesoamerican races, including races 9, 23, 31, 55, 64, 65, 81, 83, 89, 95, 449, 453, and 1545 [45,49,52]. Compared with other Andean cultivars, Jalo Vermelho shows a broader resistance spectrum, surpassing differential cultivars such as Michigan Dark Red Kidney and Perry Marrow, as well as Paloma, Jalo Listras Pretas, Pitanga, Corinthiano, Beija Flor, and Jalo EEP 558 (Table S5) [49]. In addition, its resistance to race 63-39 of *Pseudocercospora griseola*, the causal agent of angular leaf spot, further highlights its relevance as a multi-disease resistance source for breeding programs.

Race 1545 of *C. lindemuthianum*, used in this study, has been identified in Colombia, Costa Rica, Guatemala, Mexico, and Honduras, demonstrating its wide geographic distribution and epidemiological importance [53,54]. The presence of this race across diverse bean-producing regions reinforces the need to identify and characterize resistance genes effective against race 1545, as such genes are likely to contribute to broader and more durable disease control.

Previous evaluations of F_2:3_ families derived from the Jalo Vermelho × Crioulo 159 cross, inoculated with races 55 and 1545, demonstrated cosegregation of the *Co-12* gene for both races [55]. In this study, the inoculation of 172 F_2:3_ families with race 1545 resulted in the expected Mendelian segregation ratio of 1:2:1, confirming the monogenic inheritance of *Co-12*. Furthermore, genotypic analysis revealed the precise localization of *Co-12* on the upper arm of chromosome Pv04 within a 41 kb genomic interval flanked by BARCPVSSR04557 and BARCPVSSR04570. The tight linkage between *Co-12* and BARCPVSSR04557 highlights its practical value for marker-assisted selection, facilitating the development of cultivars with durable anthracnose resistance. These markers could be used in marker-assisted selection; however, they have not yet been validated in independent populations.

The upper arm of chromosome Pv04 has been reported as a genomic region containing an important cluster of genes conferring resistance to multiple pathogens. This cluster includes genes for resistance to rust (*Ur-5*, *Ur-14*, *Ur-Dorado-108*, *Ur-PI310762* and *Ur-19833*) [56,57,58,59], angular leaf spot [24,60,61], bean golden yellow mosaic virus (BGYMV) [62], and anthracnose. The presence of multiple resistance genes in this cluster highlights its potential as a key target for breeding programs aimed at developing cultivars with broad and durable resistance to different diseases.

Numerous anthracnose resistance genes from both Andean and Mesoamerican gene pools have been mapped to Pv04, including the *Co-3* allelic series (Mexico 222, Mexico 227, PI 207262, BAT 93, Ouro Negro, G2333, MDRK, Kaboon, Widusa, A 252, and Xana) [19,21,22,23,24,33,41,44,63], as well as *Co-y* and *Co-z* in Jalo EEP 558 [48], *Co-15* in Corinthiano [46], *Co-16* in Crioulo 159 [37], and *Co-Bf* in Beija Flor [51]. In addition, GWASs have identified loci associated with resistance to races 2, 4, 7, 9, 65, 81, and 109 on Pv04 [16,42,64,65,66]. This dense accumulation of resistance loci confirms that Pv04 is a central genomic region for anthracnose resistance breeding.

Allelism tests performed between Jalo Vermelho and multiple resistant cultivars demonstrated that *Co-12* is genetically independent from several resistance genes, including *Co-1*^3^, *Co-2*, *Co-3*, *Co-3*^3^, *Co-3*^M^, *Co-3*^K^, *Co-3*^W^, *Co-3*^4^, *Co-4*, *Co-4*^3^, *Co-5*, *Co-6*, *Co-7*, *Co-9*, *Co-11*, *Co-13* [45], *Co-15* [46], *Co-16* [37], and *Co-Bf* [50], as all the crosses exhibited the expected 15:1 resistance–susceptible segregation ratio. This genetic independence reinforces the importance of *Co-12* and supports its use in pyramiding strategies aimed at enhancing resistance durability.

Comparative physical mapping further supported the distinct position of *Co-12*. The *Co-3*^4^ allele, which cosegregates with the *Phg-ON* gene in Ouro Negro, is located approximately 3.2 Mb from BARCPVSSR04570 [24], whereas *Co-15* in Corinthiano lies approximately 8.9 Mb from the *Co-12* region [46]. The *Co-Bf* gene from Beija Flor is located downstream of *Co-12*, between markers SS333 and SS509 [51], representing the closest mapped resistance gene to *Co-12*. These distances confirm that *Co-12* occupies a unique genomic position within the Pv04 resistance cluster.

Within the 41 kb *Co-12* interval, three candidate genes were identified: one encoding a gamma-glutamyl-GABA protein and two encoding a lipid transfer protein (LTP2). Lipid transfer proteins are widely recognized as multifunctional components of plant defense and participate in cuticle formation, signal transduction, and direct antimicrobial activity. Several studies have demonstrated that LTPs can inhibit fungal growth, contribute to cell wall reinforcement, and modulate host–pathogen interactions. Therefore, the presence of two LTP2 genes within the *Co-12* genomic interval strongly suggests a potential role in resistance to *C. lindemuthianum*. Most disease resistance genes in plants belong to the NBS-LRR or kinase families, which are highly abundant on chromosomes Pv04, Pv10, and Pv11 in the common bean [65,67,68]. In contrast, lipid transfer proteins are less commonly associated with resistance loci, although they have long been recognized for their antifungal and antibacterial activities [69,70]. LTP-mediated defense responses have been reported in *Capsicum annuum* [71], *Brassica rapa* [72], *Triticum durum* [73], *Arabidopsis thaliana* [74], and *Gossypium hirsutum* [75].

Similarity analysis against *A. thaliana* revealed identities ranging from 44.7% to 71.9%. The gene annotated as Gamma-glutamyl-gamma-aminobutyrate hydrolase (Gamma-glutamyl-GABA) in Phytozome showed 71.9% similarity, confirming its predicted function. Genes annotated as LTP2 in Phytozome displayed moderate similarity (44.7–53.8%) to Tracheary Element Differentiation-Related 4 in Arabidopsis. Previous studies in Zinnia mesophyll cells revealed differentiation-specific genes associated with tracheary element formation that exhibit typical LTP features [76]. Notably, in Phytozome, the LTP2 genes exhibit 98.1% sequence similarity with those in *Phaseolus vulgaris* and 83.7% with those in *Phaseolus acutifolius*, suggesting that despite evolutionary divergence and differences in nomenclature, conserved domains allow inference of the likely functions of these candidate genes in the common bean.

This study represents the first report linking LTP2 genes to anthracnose resistance in *Phaseolus vulgaris*. The identification of LTP2 genes within the *Co-12* interval suggests that this resistance locus may involve a noncanonical defense mechanism, expanding the current understanding of resistance pathways in the common bean.

Although fine mapping narrowed the target region to a small genomic interval containing three candidate genes, further studies are needed to functionally validate these genes. Specifically, gene expression analyses and gene-editing approaches should be used to identify causal genes. In addition, comprehensive validation of the associated molecular markers across diverse genetic backgrounds and environments is necessary to confirm their stability and reliability for effective application in breeding programs.

## 4. Materials and Methods

### 4.1. Experimental Site and Plant Material

The experiments were conducted in a greenhouse at the Núcleo de Pesquisa Aplicada à Agricultura (Nupagri), Universidade Estadual de Maringá (UEM), Paraná, Brazil (23°26′7.744″ S, 51°53′43.288″ W), between July 2018 and December 2022. The seeds of all the cultivars used in this study were obtained from the Common Bean Germplasm Bank (BGF) of UEM.

The plant material was derived from a biparental cross between the Andean cultivar Jalo Vermelho and the Mesoamerican cultivar Crioulo 159. Crosses were performed in a greenhouse in 2018. In 2019, F_1_ plants were grown, and F_2_ seeds were subsequently harvested. In 2020, F_2_ seeds were sown to generate F_2:3_ families. When the trifoliate leaves of F_2_ plants had expanded, leaf tissue was collected for DNA extraction. The plants were maintained in the greenhouse to produce seeds, with each F_2_ plant harvested separately, generating 172 F_2:3_ families. In 2021, the F_2:3_ families were phenotyped, and F_2_ plants were genotyped using the BeadChip platform; in 2022, all F_2_ plants were genotyped with SSR markers.

To increase the number of seeds, seeds from the F_1_, F_2_, and parental plants were sown individually in plastic pots (8 × 8 × 12 cm) filled with biostabilized Pinus bark substrate (MecPlant—Registration EP PB 09549-4/MAPA Brasil, MEC PREC Ind. Com. Ltda., Telêmaco Borba, PR, Brazil) and maintained under greenhouse conditions until harvest.

### 4.2. Inoculum Preparation, Inoculation and Evaluation

The *Colletotrichum lindemuthianum* race 1545 isolate was cultured on Petri dishes containing potato-dextrose-agar medium. For inoculum production, test tubes containing bean pods (8–10 cm) partially immersed (1–2 cm) in water–agar medium were autoclaved at 120 °C for 40 min. Small fragments of the isolate race 1545 of *C. lindemuthianum* from the Petri dish were placed on the pods using a metal loop that was sterilized via flame sterilization; this procedure was carried out in a laminar flow hood. The tubes were then incubated in a growth chamber at 20 ± 2 °C for 14 days to promote sporulation [77].

After incubation, the pods containing spores were removed using sterile forceps and transferred to beakers containing autoclaved distilled water. The suspension was filtered through double-layer gauze to obtain a spore suspension. The spore concentration was adjusted to 1.2 × 10^6^ conidia mL^−1^ using a Neubauer hemocytometer, and one drop of Tween 20 was added per 100 mL of suspension [78].

For anthracnose evaluation, the F_2:3_ families were sown in plastic trays (48 × 30 × 11 cm) filled with MecPlant substrate. Each family consisted of approximately 8–10 seeds, depending on seed availability, resulting in a total of 1651 F_3_ plants evaluated phenotypically. Comparable family sizes have been successfully employed in previous studies analyzing resistance inheritance in F_2:3_ populations inoculated with *C. lindemuthianum* races, demonstrating that this sampling strategy is sufficient to detect segregation patterns and reliably assess resistance inheritance [51,58]. Six genotypes were sown per tray, with seeds spaced 8 cm apart between genotypes and 3 cm between individual seeds, including the parents, controls, and F_2:3_ families.

At the V3 growth stage, corresponding to the full expansion of the first trifoliate leaf, the trays were transferred to a fog chamber (20 ± 2 °C) for inoculation. The differential cultivars Widusa (resistant) and Cornell 49-242 (susceptible) were included as controls.

Each plant was individually inoculated with race 1545 of *C. lindemuthianum* and considered an independent biological replicate for assessing disease response. This experimental design enabled accurate estimation of the proportion of resistant and susceptible plants within each family and supported reliable genetic segregation analysis of the trait.

The plants were inoculated by spraying the suspension onto both the abaxial and adaxial surfaces of the trifoliate leaves and applying one spray per leaf on each surface. Trays were then incubated for 72 h at 20 ± 2 °C under controlled lighting (12 h light at 680 lux and 12 h darkness) and a relative humidity above 95%.

Anthracnose symptoms can be observed between 10 and 15 days after inoculation, depending on the virulence of the isolate under study. In this population, visual assessments were conducted 10 days after inoculation, as the virulence of race 1545 produced pronounced symptoms. The standard anthracnose severity scale was used, with the following scores: 1—Absence of symptoms; 2—Up to 1% of the veins show necrotic spots, noticeable only on the underside of the leaves; 3—Up to 3% of the veins show necrotic spots, noticeable only on the underside of the leaves; 4—Up to 1% of the veins show necrotic spots, noticeable on both sides of the leaves; 5—Up to 3% of the veins show necrotic spots, noticeable on both sides of the leaves; 6—Necrotic spots on the veins, noticeable on both sides of the leaves, with some lesions on stems, branches, and petioles; 7—Necrotic spots on most veins and a large portion of adjacent mesophyll tissue, which ruptured, with abundant lesions on stems, branches, and petioles; 8—Necrotic spots on almost all veins, very abundant on stems, branches, and petioles, causing ruptures, defoliation, and reduced plant growth; 9—Plant death. Scores from 1 to 3 indicate resistant plants, and scores from 4 to 9 indicate susceptible plants [79]. Families were classified as homozygous resistant when all the evaluated F_3_ plants were resistant, as susceptible when all the evaluated F_3_ plants were susceptible, and as heterozygous when the F_3_ plants showed a mix of resistant and susceptible individuals.

### 4.3. Inheritance Analysis

Previous inheritance studies have shown that Jalo Vermelho has a single dominant gene [45]. To confirm these results, the inheritance of resistance in the Jalo Vermelho cultivar was evaluated using the 172 F_2:3_ families inoculated with race 1545 of *Colletotrichum lindemuthianum*. Based on the phenotypic classification of resistant, susceptible and heterozygous families, segregation ratios were analyzed using the chi-square (χ^2^) test to verify conformity with the expected Mendelian segregation for a single dominant gene in the F_2_ population. A bar graph was constructed to better visualize the results of the chi-square test.

### 4.4. SNP Genotyping and Primary Mapping

Given that Jalo Vermelho carries a single gene, we decided to perform fine mapping by first genotyping a subset of the F_2_ plants with 5398 SNPs using the BeadChip to identify the chromosome and approximate region where the gene is located. SSR markers were subsequently designed within this specific region to genotype the entire population and further narrow the location of the gene [49,51,58,59].

Genomic DNA was extracted from 172 F_2_ plants and the parental cultivars using the PureLink Genomic Plant DNA Purification Kit (Thermo Fisher Scientific, Waltham, MA, USA), following the manufacturer’s instructions. Based on the phenotyping of the F_2:3_ families with race 1545 of *Colletotrichum lindemuthianum*, 17 resistant and 18 susceptible families were selected, and the DNA of the corresponding F_2_ progenies was sent to Soybean Genomics and Improvement Laboratory, USDA-ARS/BARC-W (Beltsville, MD, USA), for genotyping using the BARBean6K_3 Illumina BeadChip containing 5398 SNP markers on the Illumina Infinium^®^ HD Assay Ultra platform [80].

Fluorescence signals were captured with an Illumina BeadArray Reader (Illumina, Inc., San Diego, CA, USA), and allele calling was performed using GenomeStudio V2011.1 (Illumina, Inc.). All 5389 SNPs were visually inspected and manually curated when necessary. SNPs were distributed across all 11 chromosomes of the common bean; however, SNPs linked to the *Co-12* gene were identified exclusively on chromosome Pv04. SNPs were considered linked to *Co-12* when they were polymorphic between parents and when resistant F_2_ individuals clustered with the resistant parent, while susceptible individuals clustered with the susceptible parent.

### 4.5. SSR Marker Development and Genotyping

Genomic fragments containing SNPs from the BeadChip were used to develop SSR markers [58]. SNPs associated with the *Co-12* region were selected, and the corresponding SSR markers were used to genotype the 172 F_2_ individuals.

PCRs were performed with 20 µL reaction mixtures containing 50 ng of genomic DNA, 0.25 µM of each primer, PCR buffer, 1.5 mM MgCl_2_, 0.2 mM of each dNTP, Taq DNA polymerase (Invitrogen, Thermo Fisher Scientific, Waltham, MA, USA), and ultrapure water. Amplified products were separated on 6% nondenaturing polyacrylamide gels and stained with 0.02% SYBR Safe. Bands were visualized under ultraviolet light, and images were captured using an L-PIX Image EX system (Loccus Biotecnologia, Cotia, SP, Brazil).

The physical positions of SSR markers were determined by BLAST analysis against the *Phaseolus vulgaris* v2.1 reference genome using the Phytozome database: “https://phytozome-next.jgi.doe.gov/ (accessed on 14 January 2026)”.

### 4.6. Linkage Map Construction

Genetic linkage analysis was performed using JoinMap 4.0 [81]. Marker ordering and distance estimation were carried out using the regression mapping algorithm with the Kosambi mapping function. Linkage groups were established using a minimum LOD threshold of 3.0 and a maximum recombination distance of 50 cM. The final linkage map was visualized using MapChart 2.3 [82].

### 4.7. Candidate Gene Identification

The physical position of *Co-12* was determined based on the flanking SSR markers using BLAST analysis against the *Phaseolus vulgaris* v2.1 reference genome in Phytozome. All annotated genes located within the marker-defined interval were retrieved, and those with predicted functions related to plant defense or stress response were considered potential *Co-12* candidate genes.

## 5. Conclusions

The Andean common bean cultivar Jalo Vermelho represents a highly valuable source of anthracnose resistance, conferring broad effectiveness against multiple races of *Colletotrichum lindemuthianum*. The *Co-12* gene was precisely mapped to a 41 kb genomic region on chromosome Pv04, confirming its monogenic inheritance and genetic independence from previously described resistance loci within the same chromosomal cluster. The closely linked SSR markers BARCPVSSR04557 and BARCPVSSR04570 provide reliable tools for marker-assisted selection and will facilitate the efficient introgression of *Co-12* into elite breeding lines. The identification of candidate genes encoding gamma-glutamyl-GABA proteins and lipid transfer proteins suggests that *Co-12*-mediated resistance may involve a noncanonical defense mechanism distinct from that of classical NBS-LRR pathways. These findings expand current knowledge of resistance mechanisms in *Phaseolus vulgaris* and highlight the diversity of host defense strategies against anthracnose. Overall, the high-resolution mapping of *Co-12* and the development of closely linked molecular markers provide important genomic resources for common bean breeding programs and contribute directly to the development of cultivars with durable and broad-spectrum anthracnose resistance.

## Figures and Tables

**Table 1:** Reactions of 172 F_2:3_ families from the Jalo Vermelho × Crioulo 159 cross inoculated with race 1545 of *Colletotrichum lindemuthianum*.

Parental Lines and Cross	Generation	Observed Segregation (RR:RS:SS)	Expected Segregation (RR:RS:SS)	χ^2^	Probability (%)
JV	PR	10:0	10:0		
Crioulo 159	PS	0:10	0:10		
JV × Crioulo 159	F_2:3_	45:83:44	43:86:43	0.221	89.54

JV = Jalo Vermelho; PR = resistant parent; PS = susceptible parent. RR = resistant; RS = heterozygous; SS = susceptible.

**Table 2:** SSR markers associated with the *Co-12* gene (v2.1).

SSR BARC ID	Motif	Forward Primer Sequence	Reverse Primer Sequence	SSR Start Position	SSR End Position
BARCPVSSR04557	(TA)5	TCCAATTTGCG-	TGAGTCTCTTTG-	94,873	95,157
		AGAGAGTGA	CTTTGCAATTA		
BARCPVSSR04570	(TA)12	CCCCATTAAG-	TCTCAAAGACGG-	136,199	136,434
		GTAATGAAAAGC	GGCATAAC		
BARCPVSSR04574	(AT)11	ACCCCGGAAG-	CCCGATAAACTG-	269,985	270,262
		AGATTTCAAG	GAACCAAA		
BARCPVSSR04577	(AT)14	TAGAGCCACCA-	TTTATAAGCATT-	430,389	430,669
		TTGCCTTTC	TGCATTTTACATAAC		
BARCPVSSR04619	(TA)11	GGCCCAATTC-	TTAAAACCGGCA-	2,145,580	2,145,876
		TTTTTCTACCA	CTATTTGATT		

**Table 3:** Genotyping with SSR of Jalo Vermelho, Crioulo 159 and four F_2_ plants from the Jalo Vermelho × Crioulo 159 cross and their reaction to race 1545 of *Colletotrichum lindemuthianum*. The *Co-12* gene is in a 41 kb genomic region on chromosome Pv04, between the SSR4557 and SSR4570 markers.

Physical Position in Pv04 (bp)		94,873	136,199	269,985	430,389	2,145,580
F_2_	Reaction to Race 1545 of *C. lindemuthianum*	SSR	SSR	SSR	SSR	SSR
		4557	4570	4574	4577	4619
JV	Resistant	BB	BB	BB	BB	BB
C159	Susceptible	AA	AA	AA	AA	AA
111	Heterozygous	AB	AB	AA	AA	AA
113	Susceptible	AA	AB	AB	AB	AB
169	Heterozygous	AB	AA	AA	AA	AA
171	Heterozygous	AB	AA	AA	AA	AA

**Table 4:** Candidate genes within the genomic region where the *Co-12* gene was mapped between BARCPVSSR04557 and BARCPVSSR04570 SSR markers.

Candidate Gene	Physical Position (bp) Start-End	Homology in *A. thaliana*	Functional Annotation (TAIR) ^1^	Similarity	Functional Annotation (Phytozome) ^2^
Phvul.004G002800	119,700–122,418	AT5G38200	Gamma-glutamyl-gamma-aminobutyrate hydrolase/Gamma-glutamyl-GABA	71.9%	Gamma-glutamyl-GABA
Phvul.004G002700	126,825–127,577	AT3G18280	Tracheary element differentiation-related 4	44.7%	Probable lipid transfer (LTP_2)
Phvul.004G002600	129,831–130,579	AT3G18280	Tracheary element differentiation-related 4	53.8%	Probable lipid transfer (LTP_2)

^1^ Functional annotation in TAIR—The *Arabidopsis* Information Resource: “https://www.arabidopsis.org (accessed on 14 January 2026)”; ^2^ Functional annotation in Phytozome—Common bean reference genome v.2.1: “https://phytozome.jgi.doe.gov# (accessed on 14 January 2026)”.

## Data Availability

The original contributions presented in this study are included in the article/Supplementary Materials. Further inquiries can be directed to the corresponding author.

## Supplementary Materials

The following supporting information can be downloaded at https://www.mdpi.com/article/10.3390/plants15060931/s1. Table S1: Genotyping of the 17 F_2_ plants from the Jalo Vermelho × Crioulo 159 cross-resistant to race 1545 of *C. lindemuthianum*. Genotyping was performed with the BeadChip platform, which consists of 5398 SNPs, of which 150 were associated with the *Co-12* gene on chromosome Pv04; Table S2: Genotyping of the 18 F_2_ plants from the Jalo Vermelho × Crioulo 159 cross-susceptible to race 1545 of *C. lindemuthianum*. Genotyping was performed with the BeadChip platform, which consists of 5398 SNPs, of which 150 were associated with the *Co-12* gene on chromosome Pv04; Table S3: A total of 150 SNP markers associated with the *Co-12* gene on Pv04 of the common bean; Table S4: Genotyping of 172 F_2_ plants from the Jalo Vermelho × Crioulo 159 cross with five SSR markers and inoculated with race 1545 of *C. lindemuthianum*; Table S5. Reactions of common bean cultivars inoculated with Andean and Mesoamerican races of *Colletotrichum lindemuthianum*. Adapted from [49].

## Abbreviations

The following abbreviations are used in this manuscript:

SNP	Single-nucleotide polymorphism
SSR	Simple Sequence Repeats

## References

[1] Debouck D., Singh S.P. (1999). Diversity in *Phaseolus* species in relation to the common bean. Common Bean Improvement in the Twenty-First Century.

[2] Bitocchi E., Rau D., Bellucci E., Rodriguez M., Murgia M.L., Gioia T., Santo D., Nanni L., Attene G., Papa R. (2017). Beans (*Phaseolus* ssp.) as a model for understanding crop evolution. Front. Plant Sci..

[3] Gepts P., Aragão F., Barros E., Blair M., Brondani R., Broughton W., Galasso I., Hernandez G., Kami J., Lariguet P., Moore P.H., Ming R. (2008). Genomics of Phaseolus beans, a major source of dietary protein and micronutrients in the tropics. Genomics of Tropical Crop Plants.

[4] Celmeli T., Sari H., Canci H., Sari D., Adak A., Eker T., Toker C. (2018). The nutritional content of common bean (*Phaseolus vulgaris* L.) landraces in comparison to modern varieties. Agronomy.

[5] Vieira N.M., Peghinelli V.V., Monte M.G., Costa N.A., Pereira A.G., Seki M.M., Azevedo P.S., Polegato B.F., Paiva S.A.R., Zornoff L.A.M. (2023). Beans consumption can contribute to the prevention of cardiovascular disease. Clin. Nutr. ESPEN.

[6] Conab Companhia Nacional de Abastecimento. http://www.conab.gov.br.

[7] Melotto M., Kelly J.D., Sciences S., Lansing E., Melotto M., Kelly J.D. (2000). An allelic series at the *Co-1* locus conditioning resistance to anthracnose in common bean of Andean origin. Euphytica.

[8] Alzate-Marin A.L., Costa M.R., Menarim H., Moreira M.A., Barros E.G. (2003). Herança da resistência à antracnose na cultivar de feijoeiro comum Cornell 49-242. Fitopatol. Bras..

[9] Padder B.A., Sharma P.N., Awale H.E., Kelly J.D. (2017). *Colletotrichum lindemuthianum*, the causal agent of bean anthracnose. J. Plant Pathol..

[10] Nunes M.P.B.A., Gonçalves-Vidigal M.C., Martins V.S.R., Xavier L.F.S., Valentini G., Bisneta M.V., Vidigal Filho P.S. (2021). Relationship of *Colletotrichum lindemuthianum* races and resistance loci in the *Phaseolus vulgaris* L. genome. Crop Sci..

[11] Rodríguez-Guerra R., Ramirez-Rueda M.T., Martinez de la Vega O., Simpson J. (2003). Variation in genotype, pathotype and anastomosis groups of *Colletotrichum lindemuthianum* isolates from Mexico. Plant Pathol..

[12] Ferreira J.J., Campa A., Kelly J.D., Tuberosa R., Varshney R. (2013). Organization of genes conferring resistance to anthracnose in common bean. Translational Genomics for Crop Breeding.

[13] Kelly J.D., Vallejo V. (2004). A comprehensive review of the major genes conditioning resistance to anthracnose in common bean. HortScience.

[14] Pastor-Corrales M.A. (1996). Traditional and molecular confirmation of the coevolution of beans and pathogens in Latin America. Annu. Rep. Bean Improv. Coop..

[15] Kelly J.D., Young R.A. (1996). Proposed symbols for anthracnose resistance genes. Annu. Rep. Bean Improv. Coop..

[16] Vaz Bisneta M., Gonçalves-Vidigal M.C. (2020). Integration of anthracnose resistance loci and RLK and NBS-LRR-encoding genes in the *Phaseolus vulgaris* L. genome. Crop Sci..

[17] Young R.A., Kelly J.D. (1996). Characterization of the genetic resistance to *Colletotrichum lindemuthianum* in common bean differential cultivars. Plant Dis..

[18] Young R.A., Kelly J.D. (1996). RAPD markers flanking the Are gene for anthracnose resistance in common bean. J. Am. Soc. Hortic. Sci..

[19] Rodríguez-Suárez C., Méndez-Vigo B., Pañeda A., Ferreira J.J., Giraldez R. (2007). A genetic linkage map of *Phaseolus vulgaris* L. and localization of genes for specific resistance to six races of anthracnose (*Colletotrichum lindemuthianum*). Theor. Appl. Genet..

[20] Campa A., Trabanco N., Ferreira J.J. (2017). Identification of clusters that condition resistance to anthracnose in the common bean differential cultivars AB 136 and MDRK. Phytopathology.

[21] Rodríguez-Suárez C., Ferreira J.J., Campa A., Pañeda A., Giraldes R. (2008). Molecular mapping and intra-cluster recombination between anthracnose race-specific resistance genes in the common bean differential cultivars Mexico 222 and Widusa. Theor. Appl. Genet..

[22] Hallard J., Trebuchet G. (1976). Bean anthracnose in Western Europe. Annu. Rep. Bean Improv..

[23] Murube E., Campa A., Ferreira J.J. (2019). Integrating genetic and physical positions of the anthracnose resistance genes described in bean chromosomes Pv01 and Pv04. PLoS ONE.

[24] Gonçalves-Vidigal M.C., Cruz A.S., Lacanallo G.F., Vidigal Filho P.S., Sousa L.L., Pacheco C.M.N.A., McClean P., Gepts P., Pastor-Corrales M.A. (2013). Co-segregation analysis and mapping of the anthracnose *Co-10* and angular leaf spot *Phg-ON* disease-resistance genes in the common bean cultivar Ouro Negro. Theor. Appl. Genet..

[25] Young R.A., Melotto M., Nodari R.O., Kelly J.D. (1998). Marker-assisted dissection of the oligogenic anthracnose resistance in the common bean cultivar “G 2333”. Theor. Appl. Genet..

[26] Queiroz V.T., Sousa C.S., Costa M.R., Sanglad D.A., Arruda K.M.A., Souza T., Ragagnin V.A., Barros E.G., Moreira M.A. (2004). Development of SCAR markers linked to common bean anthracnose resistance genes *Co-4* and *Co-6*. Annu. Rep. Bean Improv. Coop..

[27] Alzate-Marin A.L., Menarim H., Baía G.S., Paula-Júnior T.J., Souza K.A., Costa M.R., Barros E.G., Moreira M.A. (2001). Inheritance of anthracnose resistance in the common bean differential cultivar G 2333 and identification of a new molecular marker linked to the *Co-4*^2^ gene. J. Phytopathol..

[28] Awale H.E., Kelly J.D. (2001). Development of SCAR markers linked to *Co-4*^2^ gene in common bean. Annu. Rep. Bean Improv. Coop..

[29] Melotto M., Kelly J.D., Melotto M. (2001). Fine mapping of the *Co-4* locus of common bean reveals a resistance gene candidate, *COK-4*, that encodes for a protein kinase. Theor. Appl. Genet..

[30] Oblessuc P.R., Francisco C., Melotto M. (2015). The *Co-4* locus on chromosome Pv08 contains a unique cluster of 18 COK-4 genes and is regulated by immune response in common bean. Theor. Appl. Genet..

[31] Burt A.J., William H.M., Perry G., Khanal R., Pauls K.P., Kelly J.D., Navabi A. (2015). Candidate gene identification with SNP marker-based fine mapping of anthracnose resistance gene *Co-4* in common bean. PLoS ONE.

[32] De Arruda M.C.C., Alzate-Marin A.L., Chagas J.M., Moreira M.A., Barros E.G. (2000). Identification of random amplified polymorphic DNA markers linked to the *Co-4* resistance gene to *Colletotrichum lindemuthianum* in common bean. Phytopathology.

[33] Campa A., Giraldez R., Ferreira J.J. (2009). Genetic dissection of the resistance to nine anthracnose races in the common bean differential cultivars MDRK and TU. Theor. Appl. Genet..

[34] Vallejo V.D., Kelly J.D. (2009). New insights into the anthracnose resistance of common bean landrace G 2333. Open Hortic. J..

[35] Young R.A., Kelly J.D. (1997). RAPD markers linked to three major anthracnose resistance genes in common bean. Crop Sci..

[36] Alzate-Marin A.L., Menarim H., Carvalho G.A., Paula-Júnior T.J., Barros E.G., Moreira M.A. (1999). Improved selection with newly identified RAPD markers linked to resistance gene to four pathotypes of *Colletotrichum lindemuthianum* in common bean. Phytopathology.

[37] Coimbra-Gonçalves G.K., Gonçalves-Vidigal M.C., Coelho R.T., Valentini G., Filho P.S.V., Lacanallo G.F., Elias H.T. (2016). Characterization and mapping of anthracnose resistance gene in Mesoamerican common bean cultivar Crioulo 159. Crop Sci..

[38] Trabanco N., Campa A., Ferreira J.J. (2015). Identification of a new chromosomal region involved in the genetic control of resistance to anthracnose in common bean. Plant Genome.

[39] Geffroy V., Sévignac M., Billant P., Dron M., Langin T. (2008). Resistance to *Colletotrichum lindemuthianum* in *Phaseolus vulgaris*: A case study for mapping two independent genes. Theor. Appl. Genet..

[40] Geffroy V. (1997). Dissection Génétique de la Résistance à *Colletotrichum lindemuthianum*, Agente de L’anthracnose, chez deux Génotypes Représentatifs des Pools Géniques de *Phaseolus vulgaris*. Ph.D. Thesis.

[41] Campa A., Rodríguez-Suárez C., Giraldez R., Ferreira J. (2014). Genetic analysis of the response to eleven *Colletotrichum lindemuthianum* races in a RIL population of common bean (*Phaseolus vulgaris* L.). BMC Plant Biol..

[42] Zuiderveen G.H., Padder B.A., Kamfwa K., Song Q., Kelly J.D. (2016). Genome-wide association study of anthracnose resistance in Andean beans (*Phaseolus vulgaris*). PLoS ONE.

[43] Chen M., Wu J., Wang L., Mantri N., Zhang X., Zhu Z., Wang S. (2017). Mapping and genetic structure analysis of the anthracnose resistance locus *Co-1HY* in the common bean (*Phaseolus vulgaris* L.). PLoS ONE.

[44] Campa A., Giraldez R., Ferreira J.J. (2011). Genetic analysis of the resistance to eight anthracnose races in the common bean differential cultivar Kaboon. Phytopathology.

[45] Gonçalves-Vidigal M.C., Lacanallo G.F., Vidigal Filho P.S. (2008). A new gene conferring resistance to anthracnose in Andean common bean (*Phaseolus vulgaris* L.) cultivar ‘Jalo Vermelho’. Plant Breed..

[46] Sousa L.L., Gonçalves A.O., Gonçalves-Vidigal M.C., Lacanallo G.F., Fernandez A.C., Awale H., Kelly J.D. (2015). Genetic characterization and mapping of anthracnose resistance of common bean landrace cultivar Corinthiano. Crop Sci..

[47] Richard M.S., Pflieger S., Sévignac M., Thareau V., Blanchet S., Li Y., Jackson S.A., Geffroy V. (2014). Fine mapping of *Co-x*, an anthracnose resistance gene to a highly virulent strain of *Colletotrichum lindemuthianum* in common bean. Theor. Appl. Genet..

[48] Geffroy V., Delphine S., Oliveira J.C.F., Sévignac M., Cohen S., Gepts P., Neema C., Langin T., Dron M. (1999). Identification of an ancestral resistance gene cluster involved in the coevolution process between *Phaseolus vulgaris* and its fungal pathogen *Colletotrichum lindemuthianum*. Mol. Plant-Microbe Interact..

[49] Lima Castro S.A., Gonçalves-Vidigal M.C., Gilio T.A.S., Lacanallo G.F., Valentini G., Da Silva Ramos Martins V., Pastor-Corrales M.A. (2017). Genetics and mapping of a new anthracnose resistance locus in Andean common bean Paloma. BMC Genom..

[50] Marcon J.R.S., Gonçalves-Vidigal M.C., Paulino J.F.C., Vidigal Filho P.S., Coêlho M. (2020). Genetic resistance of common bean cultivar Beija Flor to *Colletotrichum lindemuthianum*. Acta Sci. Agron..

[51] Xavier L.F.S., Valentini G., Gonçalves-Vidigal M.C., Poletine J.P., Vidigal Filho P.S., Hurtado-Gonzales O.P., Song Q., He R., Pastor-Corrales M.A. (2025). Fine mapping of the *Bf* gene conferring broad-spectrum resistance to the anthracnose pathogen of common bean (*Phaseolus vulgaris* L.). Theor Appl Genet..

[52] Vidigal Filho P.S., Gonçalves-Vidigal M.C., Kelly J.D., Kirk W.W. (2007). Sources of resistance to anthracnose in traditional common bean cultivars from Paraná, Brazil. J. Phytopathol..

[53] Balardin R.S., Jarosz M., Kelly J.D. (1997). Virulence and molecular diversity in *Colletotrichum lindemuthianum* from South, Central, and North America. Phytopathology.

[54] Mahuku G.S., Riascos J.J. (2004). Virulence and molecular diversity within *Colletotrichum lindemuthianum* isolates from Andean and Mesoamerican bean varieties and regions. Eur. J. Plant Pathol..

[55] Silva J.B., Gonçalves-Vidigal M.C., Vidigal Filho P.S., Valentini G., Xavier L.F.S., Martins V.S.R., Vaz Bisneta M. (2020). Gene *Co-12* of Jalo Vermelho cultivar conferring resistance to races 55 and 1545 of *Colletotrichum lindemuthianum*. Annu. Rep. Bean Improv..

[56] Miklas P.N., Pastor-Corrales M.A., Jung G., Coyne D.P., Kelly J.D., McClean P.E., Gepts P. (2002). Comprehensive linkage map of bean rust resistance genes. Annu. Rep. Bean Improv. Coop..

[57] Shin S.H., Song Q., Cregan P.B., Pastor-Corrales M.A. (2014). SSR DNA markers linked with broad-spectrum rust resistance in common bean discovered by bulk segregant analysis using a large set if SNP markers. Annu. Rep. Bean Improv. Coop..

[58] Valentini G., Gonçalves-Vidigal M.C., Hurtado-Gonzales O.P., Castro S.A.L., Cregan P.B., Song Q., Pastor-Corrales M.A. (2017). High-resolution mapping reveals linkage between genes in common bean cultivar Ouro Negro conferring resistance to the rust, anthracnose, and angular leaf spot diseases. Theor. Appl. Genet..

[59] Valentini G., Pastor-Corrales M.A., Hurtado-Gonzales O.P., Xavier L.F.S., Gill U., Song Q. (2025). Characterization and mapping of a rust resistance locus in the common bean landrace G19833. G3 Genes Genomes Genet..

[60] Mahuku G.S., Maria Iglesias A., Jara C. (2009). Genetics of angular leaf spot resistance in the Andean common bean accession G5686 and identification of markers linked to the resistance genes. Euphytica.

[61] Keller B., Manzanares C., Jara C., Lobaton J.D., Raatz B.S.B. (2015). Fine mapping of a major QTL controlling angular leaf spot resistance in common bean (*Phaseolus vulgaris* L.). Theor. Appl. Genet..

[62] Lopez C.E., Acosta I.F., Jara C., Pedraza F., Gaitan-Solis E., Gallego G., Beebe S., Tohme J. (2003). Identifying resistance gene analogs associated with resistances to different pathogens in common bean. Phytopathology.

[63] Alzate-Marin A.L., Souza K.A., Silva M.G.M., Oliveira E.J., Moreira M.A., Barros E.G. (2007). Genetic characterization of anthracnose resistance genes *Co-4*^3^ and *Co-9* in common bean cultivar Tlalnepantla 64 (PI 207262). Euphytica.

[64] Perseguini J.M.K.C., Oblessuc P.R., Rosa J.R.B.F., Gomes K.A., Chiorato A.F., Carbonell S.A.M., Garcia A.A.F., Vianello R.P., Benchimol-Reis L.L. (2016). Genome-wide association studies of anthracnose and angular leaf spot resistance in common bean (*Phaseolus vulgaris* L.). PLoS ONE.

[65] Wu J., Zhu J., Wang L., Wang S. (2017). Genome-wide association study identifies NBS-LRR-encoding genes related with anthracnose and common bacterial blight in the common bean. Front. Plant Sci..

[66] Costa L.C., Nalin R.S., Dias M.A., Ferreira M.E., Song Q., Pastor-Corrales M.A., Hurtado-Gonzales O.P., Souza E.A. (2021). Different loci control resistance to different isolates of the same race of *Colletotrichum lindemuthianum* in common bean. Theor. Appl. Genet..

[67] Meyers B.C., Kaushik S., Nandety R.S. (2005). Evolving disease resistance genes. Curr. Opin. Plant Biol..

[68] Boller T., Felix G. (2009). A renaissance of elicitors: Perception of microbe-associated molecular patterns and danger signals by pattern-recognition receptors. Annu. Rev. Plant Biol..

[69] Molina A., Segura A., Garcia-Olmedo F. (1993). Lipid transfer proteins (nsLTPs) from barley and maize leaves are potent inhibitors of bacterial and fungal plant pathogens. FEBS Lett..

[70] Cammue B.P., Thevissen K., Hendriks M., Eggermont K., Goderis I.J., Proost P., Van Damme J., Osborn R.W., Guerbette F., Kader J.C. (1995). A potent antimicrobial protein from onion seeds showing sequence homology to plant lipid transfer proteins. Plant Physiol..

[71] Diz M.S., Carvalho A.O., Ribeiro S.F., Da C.M., Beltramini L., Rodrigues R., Nascimento V.V., Machado O.L.T., Gomes V.M. (2011). Characterisation, immunolocalisation and antifungal activity of a lipid transfer protein from chili pepper (*Capsicum annuum*) seeds with novel α-amylase inhibitory properties. Physiol. Plant..

[72] Schmitt A.J., Sathoff A.E., Holl C., Bauer B., Samac D.A., Carter C.J. (2018). The major nectar protein of *Brassica rapa* is a non-specific lipid transfer protein, BrLTP2.1, with strong antifungal activity. J. Exp. Bot..

[73] Hsouna A.B., Ben S.R., Dhifi W., Mnif W., Brini F. (2021). Novel nonspecific lipid-transfer protein (TdLTP4) isolated from durum wheat: Antimicrobial activities and anti-inflammatory properties in lipopolysaccharide (LPS)-stimulated RAW 264.7 macrophages. Microb. Pathog..

[74] McLaughlin J.E., Darwish N.I., Garcia-Sanchez J., Tyagi N., Trick H.N., McCormick S., Dill-Macky R., Tumer N.E. (2021). A lipid transfer protein has antifungal and antioxidant activity and suppresses Fusarium head blight disease and DON accumulation in transgenic wheat. Phytopathology.

[75] Chen B., Zhang Y., Sun Z., Liu Z., Zhang D., Yang J., Wang G., Wu J., Ke H., Meng C. (2021). Tissue-specific expression of GhnsLTPs identified via GWAS sophisticatedly coordinates disease and insect resistance by regulating metabolic flux redirection in cotton. Plant J..

[76] Ye Z.H., Varner J.E. (1993). Gene Expression Patterns Associated with in Vitro Tracheary Element Formation in lsolated Single Mesophyll Cells of Zinnia elegans. Plant Physiol..

[77] Cárdenas F., Adams M.W., Andersen A. (1964). The genetic system for reaction of field beans (*Phaseolus vulgaris* L.) to infection by three physiologic races of *Colletotrichum lindemuthianum*. Euphytica.

[78] Pastor-Corrales M.A., López M., Fernández F., Van Schoonhoven A. (1985). Técnicas, materiales y métodos utilizados en la evaluación de frijol por su reacción a las enfermedades. Frijol: Investigación y Producción.

[79] Van Schoonhoven A., Pastor-Corrales M.A., Van Schoonhoven A., Pastor-Corrales M.A. (1987). Anthracnose. Standard System for the Evaluation of Bean Germplasm.

[80] Song Q., Jia G., Hyten D.L., Jenkins J., Hwang E.Y., Schroeder S.G., Osorno J.M., Schmutz J., Jackson S.A., McClean P.E. (2015). SNP assay development for linkage map construction, anchoring whole genome sequence and other genetic and genomic applications in common bean. G3 Genes Genomes Genet..

[81] Van Ooijen J.W. (2006). JoinMap 4, Software for the Calculation of Genetic Linkage Maps in Experimental Populations.

[82] Voorrips E.R. (2002). MapChart: Software para a apresentação gráfica dos mapas de ligação e QTLs. J. Hered..

