# Supplementary material for: Fine Mapping of the Co-12 Anthracnose Resistance Gene in the Andean Common Bean Cultivar in Brazil
Source: Plants (Basel). 2026 Mar 18;15(6):931. doi: 10.3390/plants15060931 (PMC13030795; doi:10.3390/plants15060931)
Supplement: Supplementary file 1 [file plants-15-00931-s001.zip › plants-4177286-Table S1.pdf]

Table S1: Genotyping of the 17 F_2 plants from the Jalo Vermelho × Crioulo 159 cross-resistant to race 1545 of *C. lindemuthianum* . Genotyping was performed with the BeadChip platform, which consists of 5,398 SNPs, of which 150 were associated with the *Co-12* gene on chromosome Pv04.

NCBI ss# id	Chromosome	Position (bp)	Jalo Vermelho	Crioulo 159	Resistant F_2 plants in response to *C. lindemuthianum* race 1545																
					1	2	3	4	5	6	7	8	9	10	11	12	13	14	15	16	17
715647987	3	49728839	BB	AA	AA	AB	BB	AA	AB	AB	AB	BB	AA	AA	AB	AB	AB	AB	BB	AA	AB
715646086	3	51720347	BB	AA	AA	AA	AB	AA	AA	AB	AB	BB	AA	AA	AB	AB	AB	AB	BB	AA	AB
715646083	3	51861822	BB	AA	AA	AA	AB	AA	AB	AB	AB	BB	AA	AB	AB	AB	AB	AB	BB	AB	AB
715649768	4	11168	BB	AA	BB	BB	BB	BB	BB	BB	BB	BB	BB	BB	BB	BB	BB	BB	BB	BB	BB
715649779	4	25475	BB	AA	BB	BB	BB	BB	BB	BB	BB	BB	BB	BB	BB	BB	BB	BB	BB	BB	BB
715649778	4	33308	BB	AA	BB	BB	BB	BB	BB	BB	BB	BB	BB	BB	BB	BB	BB	BB	BB	BB	BB
715649777	4	46027	BB	AA	BB	BB	BB	BB	BB	BB	BB	BB	BB	BB	BB	BB	BB	BB	BB	BB	BB
715649776	4	55042	BB	AA	BB	BB	BB	BB	BB	BB	BB	BB	BB	BB	BB	BB	BB	BB	BB	BB	BB
715649774	4	70941	BB	AA	BB	BB	BB	BB	BB	BB	BB	BB	BB	BB	BB	BB	BB	BB	BB	BB	BB
715649773	4	80183	BB	AA	BB	BB	BB	BB	BB	BB	BB	BB	BB	BB	BB	BB	BB	BB	BB	BB	BB
715649772	4	90666	BB	AA	BB	BB	BB	BB	BB	BB	BB	BB	BB	BB	BB	BB	BB	BB	BB	BB	BB
715649771	4	96165	BB	AA	BB	BB	BB	BB	BB	BB	BB	BB	BB	BB	BB	BB	BB	BB	BB	BB	BB
715649770	4	101676	BB	AA	BB	BB	BB	BB	BB	BB	BB	BB	BB	BB	BB	BB	BB	BB	BB	BB	BB
715640025	4	212864	BB	AA	BB	BB	BB	BB	BB	BB	BB	BB	BB	BB	BB	BB	BB	BB	BB	BB	BB
715648682	4	218204	BB	AA	BB	BB	BB	BB	BB	BB	BB	BB	BB	--	BB	--	BB	BB	BB	BB	BB
715640024	4	226600	BB	AA	BB	BB	BB	BB	BB	BB	BB	BB	BB	BB	BB	BB	BB	BB	BB	BB	BB
715648681	4	227060	BB	AA	BB	BB	BB	BB	BB	BB	BB	BB	BB	BB	BB	BB	BB	BB	BB	BB	BB
715648680	4	236736	BB	AA	BB	BB	BB	BB	BB	BB	BB	BB	BB	BB	BB	BB	BB	BB	BB	BB	BB
715640023	4	261173	BB	AA	BB	BB	BB	BB	BB	BB	BB	BB	BB	BB	BB	BB	BB	BB	BB	BB	BB
715648679	4	262191	BB	AA	BB	BB	BB	BB	BB	BB	BB	BB	BB	BB	BB	BB	BB	BB	BB	BB	BB
715648686	4	354682	BB	AA	BB	BB	BB	BB	BB	BB	BB	BB	BB	BB	BB	--	BB	BB	BB	BB	BB
715648683	4	381360	BB	AA	BB	BB	BB	BB	BB	BB	BB	BB	BB	BB	BB	BB	BB	BB	BB	BB	BB
715642306	4	447225	BB	AA	BB	BB	BB	BB	BB	BB	BB	BB	BB	BB	BB	BB	BB	BB	BB	BB	BB
715649432	4	532254	BB	AA	BB	BB	BB	BB	BB	BB	BB	BB	BB	BB	BB	BB	BB	BB	BB	BB	BB
715649433	4	547509	BB	AA	BB	BB	BB	BB	BB	BB	BB	BB	BB	BB	BB	BB	BB	BB	BB	BB	BB
715649434	4	554477	BB	AA	BB	BB	BB	BB	BB	BB	BB	BB	BB	BB	BB	BB	BB	BB	BB	BB	BB
715649425	4	580673	BB	AA	BB	BB	BB	BB	BB	BB	BB	BB	BB	BB	BB	BB	BB	BB	BB	BB	BB
715649427	4	593836	BB	AA	BB	BB	BB	BB	BB	BB	BB	BB	BB	BB	BB	--	BB	BB	BB	BB	BB
715639414	4	1036037	BB	AA	BB	BB	BB	BB	BB	BB	BB	BB	BB	BB	BB	BB	BB	BB	BB	BB	BB
715646904	4	1038110	BB	AA	BB	BB	BB	BB	BB	BB	BB	BB	BB	BB	BB	BB	BB	BB	BB	BB	BB
715646916	4	1086023	BB	AA	BB	BB	BB	BB	BB	BB	BB	BB	BB	BB	BB	BB	BB	BB	BB	BB	BB
715646888	4	1126285	BB	AA	BB	BB	BB	BB	BB	BB	BB	BB	BB	BB	BB	BB	BB	BB	BB	BB	BB
715646889	4	1134467	BB	AA	BB	BB	BB	BB	BB	BB	BB	BB	BB	BB	BB	BB	BB	BB	BB	BB	BB
715646891	4	1147939	BB	AA	BB	BB	BB	BB	BB	BB	BB	BB	BB	BB	BB	BB	BB	BB	BB	BB	BB
715646896	4	1224240	BB	AA	BB	BB	BB	BB	BB	BB	BB	BB	BB	BB	BB	BB	BB	BB	BB	BB	BB
715646898	4	1270861	BB	AA	BB	BB	BB	BB	BB	BB	BB	BB	BB	--	BB	--	BB	BB	BB	BB	BB
715646899	4	1278699	BB	AA	BB	BB	BB	BB	BB	BB	BB	BB	BB	BB	BB	BB	BB	BB	BB	BB	BB
715639413	4	1386459	BB	AA	BB	BB	BB	BB	BB	BB	BB	BB	BB	BB	--	BB	BB	--	BB	BB	BB
715646903	4	1433996	BB	AA	BB	BB	BB	BB	BB	BB	BB	BB	BB	BB	BB	BB	BB	BB	BB	BB	BB
715646909	4	1497419	BB	AA	BB	BB	BB	BB	BB	BB	BB	BB	BB	BB	BB	BB	BB	BB	BB	BB	BB
715646910	4	1503482	BB	AA	BB	BB	BB	BB	BB	BB	BB	BB	BB	BB	BB	BB	BB	BB	BB	BB	BB
715649973	4	1575721	BB	AA	BB	BB	BB	BB	BB	BB	BB	BB	BB	BB	BB	BB	BB	BB	BB	BB	BB
715649971	4	1627690	BB	AA	BB	BB	BB	BB	BB	BB	BB	BB	BB	BB	BB	BB	BB	BB	BB	BB	BB
715647820	4	1721502	BB	AA	BB	BB	BB	BB	BB	BB	BB	BB	BB	BB	BB	BB	BB	BB	BB	BB	BB
715647821	4	1728453	BB	AA	BB	BB	BB	BB	BB	BB	BB	BB	BB	BB	BB	BB	BB	BB	BB	BB	BB

715647822	4	1739357	BB	AA	BB	BB	BB	BB	BB	BB	BB	BB	BB	BB	BB	BB	BB	BB	BB	BB	BB
715647823	4	1745263	BB	AA	BB	BB	BB	BB	BB	BB	BB	BB	BB	BB	BB	BB	BB	BB	BB	BB	BB
715647806	4	1827663	BB	AA	BB	BB	BB	BB	BB	BB	BB	BB	BB	BB	BB	BB	BB	BB	BB	BB	BB
715647807	4	1833878	BB	AA	BB	BB	BB	BB	BB	BB	BB	BB	BB	BB	BB	BB	BB	BB	BB	BB	BB
715647808	4	1845589	BB	AA	BB	BB	BB	BB	BB	BB	BB	BB	BB	BB	BB	BB	BB	BB	BB	BB	BB
715647811	4	1865273	BB	AA	BB	BB	BB	BB	BB	BB	BB	BB	BB	--	BB	--	BB	BB	BB	BB	BB
715647813	4	1889393	BB	AA	BB	BB	BB	BB	BB	BB	BB	BB	BB	BB	BB	BB	BB	BB	BB	BB	BB
715647819	4	1982297	BB	AA	BB	BB	BB	BB	BB	BB	BB	BB	BB	BB	BB	BB	BB	BB	BB	BB	BB
715650013	4	2000276	BB	AA	BB	BB	BB	BB	BB	BB	BB	BB	BB	BB	BB	BB	BB	BB	BB	BB	BB
715650008	4	2007584	BB	AA	BB	BB	BB	BB	BB	BB	BB	BB	BB	BB	BB	BB	BB	BB	BB	BB	BB
715650009	4	2013445	BB	AA	BB	BB	BB	BB	BB	BB	BB	BB	BB	BB	BB	BB	BB	BB	BB	BB	BB
715650010	4	2026101	BB	AA	BB	BB	BB	BB	BB	BB	BB	BB	BB	BB	BB	BB	BB	BB	BB	BB	BB
715650014	4	2059126	BB	AA	BB	BB	BB	BB	BB	BB	BB	BB	BB	BB	BB	BB	BB	BB	BB	BB	BB
715650015	4	2064654	BB	AA	BB	BB	BB	BB	BB	BB	BB	BB	BB	BB	BB	BB	BB	BB	BB	BB	BB
715650016	4	2072060	BB	AA	BB	BB	BB	BB	BB	BB	BB	BB	BB	BB	BB	BB	BB	BB	BB	BB	BB
715646249	4	2136250	BB	AA	BB	BB	BB	BB	BB	BB	BB	BB	BB	BB	BB	--	BB	BB	BB	BB	BB
715646248	4	2142286	BB	AA	BB	BB	BB	BB	BB	BB	BB	BB	BB	BB	BB	BB	BB	BB	BB	BB	BB
715646246	4	2153405	BB	AA	BB	BB	BB	BB	BB	BB	BB	BB	BB	BB	BB	BB	BB	BB	BB	BB	BB
715646239	4	2276976	BB	AA	BB	BB	BB	BB	BB	BB	BB	BB	BB	BB	BB	BB	BB	BB	BB	BB	BB
715646218	4	2608903	BB	AA	BB	BB	BB	BB	BB	BB	BB	BB	BB	BB	BB	BB	BB	BB	BB	BB	BB
715646215	4	2670845	BB	AA	BB	BB	BB	BB	BB	BB	BB	BB	BB	BB	BB	BB	BB	BB	BB	BB	BB
715646214	4	2676413	BB	AA	BB	BB	BB	BB	BB	BB	BB	BB	BB	BB	BB	BB	BB	BB	BB	BB	BB
715646204	4	2758051	BB	AA	BB	BB	BB	BB	BB	BB	BB	BB	BB	BB	BB	BB	BB	BB	BB	BB	BB
715646200	4	2780607	BB	AA	BB	BB	BB	BB	BB	BB	BB	BB	BB	BB	BB	BB	BB	BB	BB	BB	BB
715646233	4	2854887	BB	AA	BB	BB	BB	BB	BB	BB	BB	BB	BB	BB	BB	BB	BB	BB	BB	BB	BB
715646228	4	2862612	BB	AA	BB	BB	BB	BB	BB	BB	BB	BB	BB	BB	BB	BB	BB	BB	BB	BB	BB
715646217	4	2880210	BB	AA	BB	BB	BB	BB	BB	BB	BB	BB	BB	BB	BB	BB	BB	BB	BB	BB	BB
715646213	4	2886459	BB	AA	BB	BB	BB	BB	BB	BB	BB	BB	BB	BB	BB	BB	BB	BB	BB	BB	BB
715646201	4	2894249	BB	AA	BB	BB	BB	BB	BB	BB	BB	BB	BB	BB	BB	BB	BB	BB	BB	BB	BB
715646224	4	2903307	BB	AA	BB	BB	BB	BB	BB	BB	BB	BB	BB	--	BB	BB	BB	BB	BB	BB	BB
715649002	4	2966427	BB	AA	BB	BB	BB	BB	BB	BB	BB	BB	BB	BB	BB	BB	BB	BB	BB	BB	BB
715649001	4	2981879	BB	AA	BB	BB	BB	BB	BB	BB	BB	BB	BB	BB	BB	BB	BB	BB	BB	BB	BB
715649010	4	3006244	BB	AA	BB	BB	BB	BB	BB	BB	BB	BB	BB	BB	BB	BB	BB	BB	BB	BB	BB
715649009	4	3018176	BB	AA	BB	BB	BB	BB	BB	BB	BB	BB	BB	BB	BB	BB	BB	BB	BB	BB	BB
715649008	4	3027795	BB	AA	BB	BB	BB	BB	BB	BB	BB	BB	BB	BB	BB	BB	BB	BB	BB	BB	BB
715649007	4	3041884	BB	AA	BB	BB	BB	BB	BB	BB	BB	BB	BB	BB	BB	BB	BB	BB	BB	BB	BB
715649006	4	3054966	BB	AA	BB	BB	BB	BB	BB	BB	BB	BB	BB	BB	BB	BB	BB	BB	BB	BB	BB
715647287	4	3085453	BB	AA	BB	BB	BB	BB	BB	BB	BB	BB	BB	BB	BB	BB	BB	BB	BB	BB	BB
715647293	4	3135346	BB	AA	BB	BB	BB	BB	BB	BB	BB	BB	BB	BB	BB	BB	BB	BB	BB	BB	BB
715647294	4	3143476	BB	AA	BB	BB	BB	BB	BB	BB	BB	BB	BB	BB	BB	BB	BB	BB	BB	BB	BB
715647284	4	3170316	BB	AA	BB	BB	BB	BB	BB	BB	BB	BB	BB	BB	BB	BB	BB	BB	BB	BB	BB
715647285	4	3176573	BB	AA	BB	BB	BB	BB	BB	BB	BB	BB	BB	BB	BB	BB	BB	BB	BB	BB	BB
715647286	4	3199273	BB	AA	BB	BB	BB	BB	BB	BB	BB	BB	BB	BB	BB	BB	BB	BB	BB	BB	BB
715650368	4	3254820	BB	AA	BB	BB	BB	BB	BB	BB	BB	BB	BB	--	BB	BB	BB	BB	BB	BB	BB
715650366	4	3279111	BB	AA	BB	BB	BB	BB	BB	BB	BB	BB	BB	BB	BB	BB	BB	BB	BB	BB	BB
715650365	4	3315474	BB	AA	BB	BB	BB	BB	BB	BB	BB	BB	BB	BB	BB	BB	BB	BB	BB	BB	BB
715650370	4	3331454	BB	AA	BB	BB	BB	BB	BB	BB	BB	BB	BB	BB	BB	BB	BB	BB	BB	BB	BB
715646816	4	3492292	BB	AA	BB	BB	BB	BB	BB	BB	BB	BB	BB	BB	BB	BB	BB	BB	BB	BB	BB
715646812	4	3523277	BB	AA	BB	BB	BB	BB	BB	BB	BB	BB	BB	BB	BB	BB	BB	BB	BB	BB	BB

715646811	4	3532372	BB	AA	BB	BB	BB	BB	BB	BB	BB	BB	BB	BB	BB	BB	BB	BB	BB	BB	BB
715646810	4	3544877	BB	AA	BB	BB	BB	BB	BB	BB	BB	BB	BB	BB	BB	BB	BB	BB	BB	BB	BB
715646807	4	3575468	BB	AA	BB	BB	BB	BB	BB	BB	BB	BB	BB	BB	BB	BB	BB	BB	BB	BB	BB
715649005	4	3715072	BB	AA	BB	BB	BB	BB	BB	BB	BB	BB	BB	BB	BB	BB	BB	BB	BB	BB	BB
715646796	4	3768718	BB	AA	BB	BB	BB	BB	BB	BB	BB	BB	BB	BB	BB	BB	BB	BB	BB	BB	BB
715646795	4	3778168	BB	AA	BB	BB	BB	BB	BB	BB	BB	BB	BB	BB	BB	BB	BB	BB	BB	BB	BB
715646793	4	3797196	BB	AA	BB	BB	BB	BB	BB	BB	BB	BB	BB	BB	BB	BB	BB	BB	BB	BB	BB
715646792	4	3830179	BB	AA	BB	BB	BB	BB	BB	BB	BB	BB	BB	BB	BB	BB	BB	BB	BB	BB	BB
715646790	4	3840559	BB	AA	BB	BB	BB	BB	BB	BB	BB	BB	BB	BB	BB	--	BB	BB	BB	BB	BB
715646788	4	3847808	BB	AA	BB	BB	BB	BB	BB	BB	BB	BB	BB	BB	BB	BB	BB	BB	BB	BB	BB
715646784	4	3905677	BB	AA	BB	BB	BB	BB	BB	BB	BB	BB	BB	BB	BB	BB	BB	BB	BB	BB	BB
715646782	4	3937414	BB	AA	BB	BB	BB	BB	BB	BB	BB	BB	BB	BB	BB	BB	BB	BB	BB	BB	BB
715648122	4	4181353	BB	AA	BB	BB	BB	BB	BB	BB	BB	BB	BB	BB	BB	BB	BB	BB	BB	BB	BB
715648121	4	4192584	BB	AA	BB	BB	BB	BB	BB	BB	BB	BB	BB	BB	BB	BB	BB	BB	BB	BB	BB
715648117	4	4260101	BB	AA	BB	BB	BB	BB	BB	BB	BB	BB	BB	BB	BB	BB	BB	BB	BB	BB	BB
715648116	4	4274980	BB	AA	BB	BB	BB	BB	BB	BB	BB	BB	BB	BB	BB	BB	BB	BB	BB	BB	BB
715648115	4	4286405	BB	AA	BB	BB	BB	BB	BB	BB	BB	BB	BB	BB	BB	BB	BB	BB	BB	BB	BB
715648114	4	4303204	BB	AA	BB	BB	BB	BB	BB	BB	BB	BB	BB	BB	BB	BB	BB	BB	BB	BB	BB
715639785	4	4308684	BB	AA	BB	BB	BB	BB	--	BB	BB	BB	BB	BB	BB	BB	BB	BB	BB	BB	BB
715648126	4	4342456	BB	AA	BB	BB	BB	BB	BB	BB	BB	BB	BB	BB	BB	BB	BB	BB	BB	BB	BB
715649532	4	4410410	BB	AA	BB	BB	BB	BB	BB	BB	BB	BB	BB	BB	BB	BB	BB	BB	BB	BB	BB
715640556	4	4429048	BB	AA	BB	BB	BB	BB	BB	BB	BB	BB	BB	BB	BB	BB	BB	BB	BB	BB	BB
715649533	4	4489669	BB	AA	BB	BB	BB	BB	BB	BB	BB	BB	BB	BB	BB	BB	BB	BB	BB	BB	BB
715649528	4	4497307	BB	AA	BB	BB	BB	BB	BB	BB	BB	BB	BB	BB	BB	BB	BB	BB	BB	BB	BB
715640555	4	4509382	BB	AA	BB	BB	BB	BB	BB	BB	BB	BB	BB	BB	BB	--	BB	BB	BB	BB	BB
715643858	4	4563346	BB	AA	BB	BB	BB	BB	BB	BB	BB	BB	BB	BB	BB	BB	BB	BB	BB	BB	BB
715647594	4	4629011	BB	AA	BB	BB	BB	BB	BB	BB	BB	BB	BB	BB	BB	BB	BB	BB	BB	BB	BB
715647600	4	4671016	BB	AA	BB	BB	BB	BB	BB	BB	BB	BB	BB	BB	BB	BB	BB	BB	BB	BB	BB
715647601	4	4679535	BB	AA	BB	BB	BB	BB	BB	BB	BB	BB	BB	BB	BB	BB	BB	BB	BB	BB	BB
715647590	4	4717287	BB	AA	BB	BB	BB	BB	BB	BB	BB	BB	BB	--	BB	--	BB	BB	BB	BB	BB
715647591	4	4730031	BB	AA	BB	BB	BB	BB	BB	BB	BB	BB	BB	BB	BB	BB	BB	BB	BB	BB	BB
715647592	4	4753192	BB	AA	BB	BB	BB	BB	BB	BB	BB	BB	BB	BB	BB	BB	BB	BB	BB	BB	BB
715639594	4	4988501	BB	AA	BB	BB	BB	BB	--	BB	BB	BB	BB	BB	BB	--	BB	BB	BB	BB	BB
715648332	4	5083634	BB	AA	BB	BB	BB	BB	BB	BB	BB	BB	BB	BB	BB	BB	BB	BB	BB	BB	BB
715648318	4	5109467	BB	AA	BB	BB	BB	BB	BB	BB	BB	BB	BB	--	BB	--	BB	BB	BB	BB	BB
715648320	4	5160162	BB	AA	BB	BB	BB	BB	BB	BB	BB	BB	BB	BB	BB	BB	BB	BB	BB	BB	BB
715648327	4	5236970	BB	AA	BB	BB	BB	BB	BB	BB	BB	BB	BB	BB	BB	BB	BB	BB	BB	BB	BB
715648328	4	5246330	BB	AA	BB	BB	BB	BB	BB	BB	BB	BB	BB	BB	BB	BB	BB	BB	BB	BB	BB
715649296	4	5489750	BB	AA	BB	BB	BB	BB	BB	BB	BB	BB	BB	BB	BB	BB	BB	BB	BB	BB	BB
715648226	4	5625817	BB	AA	BB	BB	BB	BB	BB	BB	BB	BB	BB	BB	BB	BB	BB	BB	BB	BB	BB
715648228	4	5657565	BB	AA	BB	BB	BB	BB	BB	BB	BB	BB	BB	BB	BB	BB	BB	BB	BB	BB	BB
715648222	4	5700111	BB	AA	BB	BB	BB	BB	BB	BB	BB	BB	BB	BB	BB	BB	BB	BB	BB	BB	BB
715641933	4	5975931	BB	AA	BB	BB	BB	BB	BB	BB	BB	BB	BB	BB	BB	BB	BB	BB	BB	BB	BB
715641932	4	5978333	BB	AA	BB	BB	BB	BB	BB	BB	BB	BB	BB	BB	BB	BB	BB	BB	BB	BB	BB
715641934	4	5995544	BB	AA	BB	BB	BB	BB	BB	BB	BB	BB	BB	BB	BB	BB	BB	BB	BB	BB	BB
715640044	4	6157894	BB	AA	BB	BB	BB	BB	BB	BB	BB	BB	BB	BB	BB	BB	BB	BB	BB	BB	BB
715640046	4	6241077	BB	AA	BB	BB	BB	BB	BB	BB	BB	BB	BB	BB	BB	BB	BB	BB	BB	BB	BB
715641595	4	6493282	BB	AA	BB	BB	BB	BB	BB	BB	BB	BB	BB	BB	BB	BB	BB	BB	BB	BB	BB
715644605	4	6540165	BB	AA	BB	BB	BB	BB	--	BB	BB	BB	BB	--	BB	--	BB	BB	BB	BB	BB

715644065	4	6915759	BB	AA	BB	BB	BB	BB	BB	BB	BB	BB	BB	BB	BB	BB	BB	BB	BB	BB	BB
715649153	4	7188634	BB	AA	BB	BB	BB	BB	BB	BB	BB	BB	BB	BB	BB	BB	BB	BB	BB	BB	BB
715650214	4	7366314	BB	AA	BB	BB	BB	BB	BB	BB	BB	BB	BB	BB	BB	BB	BB	BB	BB	BB	BB
715650213	4	7392047	BB	AA	BB	BB	BB	BB	BB	BB	BB	BB	BB	BB	BB	BB	BB	BB	BB	BB	BB
715639841	4	7708046	BB	AA	BB	BB	BB	BB	BB	BB	BB	BB	BB	BB	BB	BB	BB	BB	BB	BB	BB
715639839	4	7752685	BB	AA	BB	BB	BB	BB	BB	BB	BB	BB	BB	BB	BB	BB	BB	BB	BB	BB	BB
715650237	4	7965685	BB	AA	BB	BB	BB	BB	BB	BB	BB	BB	BB	BB	BB	BB	BB	BB	BB	BB	BB
715639598	4	8543342	BB	AA	BB	BB	BB	BB	BB	BB	BB	BB	BB	BB	BB	BB	BB	BB	BB	BB	BB
715639283	4	8941660	BB	AA	BB	BB	BB	BB	BB	BB	BB	BB	BB	BB	BB	BB	BB	BB	BB	BB	BB
715639280	4	9107757	BB	AA	BB	BB	BB	BB	BB	BB	BB	BB	BB	BB	BB	BB	BB	BB	BB	BB	BB
715646644	4	9259094	BB	AA	BB	BB	BB	BB	BB	BB	BB	BB	BB	BB	BB	BB	BB	BB	BB	BB	BB
715639418	4	10934677	BB	AA	BB	BB	BB	AB	AB	BB	BB	BB	BB	AB	BB	AB	BB	BB	BB	BB	BB
715640495	4	11297587	BB	AA	BB	BB	AB	AB	AB	BB	BB	BB	BB	AB	BB	AB	BB	BB	BB	BB	BB
715651182	4	11640123	BB	AA	BB	BB	AB	AB	AB	BB	BB	BB	BB	AB	BB	AB	BB	BB	BB	BB	BB
715639612	4	11694798	BB	AA	BB	BB	AB	AB	AB	BB	BB	BB	BB	AB	BB	AB	BB	BB	BB	BB	BB
715644915	4	11907429	BB	AA	BB	BB	AA	AA	AA	BB	BB	BB	BB	AA	BB	AA	BB	BB	BB	BB	BB
715640742	4	12087888	BB	AA	BB	BB	AB	AB	AB	BB	BB	BB	BB	AB	BB	AB	BB	BB	BB	BB	BB
715639838	4	12131634	BB	AA	BB	BB	AB	AB	AB	BB	BB	BB	BB	AB	BB	AB	BB	BB	BB	BB	BB

BB = resistant; AA = susceptible; AB = heterozygous; -- = missing data

Evaluation was conducted using the severity scale by van Schoonhoven and Pastor-Corrales (1987).
