# Supplementary material for: Fine Mapping of the Co-12 Anthracnose Resistance Gene in the Andean Common Bean Cultivar in Brazil
Source: Plants (Basel). 2026 Mar 18;15(6):931. doi: 10.3390/plants15060931 (PMC13030795; doi:10.3390/plants15060931)
Supplement: Supplementary file 1 [file plants-15-00931-s001.zip › plants-4177286-Table S3.pdf]

Table S3: A total of 150 SNP markers associated with the *Co-12* gene on Pv04 of the common bean.

BARCBEAN6K_3 SNP ID	NCBI ss# ID	Position (bp) (v1.0)	Position (bp) (v2.1)
BARCPV_1.0_Chr04_11168_T_G	ss715649768	11168	1695
BARCPV_1.0_Chr04_25475_G_A	ss715649779	25475	98437
BARCPV_1.0_Chr04_33308_C_T	ss715649778	33308	90604
BARCPV_1.0_Chr04_46027_C_T	ss715649777	46027	77170
BARCPV_1.0_Chr04_55042_T_C	ss715649776	55042	68415
BARCPV_1.0_Chr04_70941_G_T	ss715649774	70941	52516
BARCPV_1.0_Chr04_80183_T_G	ss715649773	80183	43274
BARCPV_1.0_Chr04_90666_G_T	ss715649772	90666	33578
BARCPV_1.0_Chr04_96165_C_T	ss715649771	96165	28079
BARCPV_1.0_Chr04_101676_G_T	ss715649770	101676	#N/A
BARCPV_1.0_Chr04_212864_A_G	ss715640025	212864	169725
BARCPV_1.0_Chr04_218204_G_A	ss715648682	218204	164385
BARCPV_1.0_Chr04_226600_G_T	ss715640024	226600	155925
BARCPV_1.0_Chr04_227060_T_C	ss715648681	227060	155465
BARCPV_1.0_Chr04_236736_G_A	ss715648680	236736	145789
BARCPV_1.0_Chr04_261173_A_G	ss715640023	261173	121346
BARCPV_1.0_Chr04_262191_C_T	ss715648679	262191	120328
BARCPV_1.0_Chr04_354682_G_A	ss715648686	354682	275483
BARCPV_1.0_Chr04_381360_T_C	ss715648683	381360	302160
BARCPV_1.0_Chr04_447225_T_C	ss715642306	447225	373157
BARCPV_1.0_Chr04_532254_C_A	ss715649432	532254	473538
BARCPV_1.0_Chr04_547509_T_C	ss715649433	547509	488793
BARCPV_1.0_Chr04_554477_A_G	ss715649434	554477	495761
BARCPV_1.0_Chr04_580673_C_A	ss715649425	580673	521957
BARCPV_1.0_Chr04_593836_G_A	ss715649427	593836	535120
BARCPV_1.0_Chr04_1036037_T_C	ss715639414	1036037	1299082
BARCPV_1.0_Chr04_1038110_T_C	ss715646904	1038110	1301156
BARCPV_1.0_Chr04_1086023_A_G	ss715646916	1086023	1901167
BARCPV_1.0_Chr04_1126285_G_A	ss715646888	1126285	1942198
BARCPV_1.0_Chr04_1134467_G_A	ss715646889	1134467	1950380
BARCPV_1.0_Chr04_1147939_A_C	ss715646891	1147939	1963852
BARCPV_1.0_Chr04_1224240_C_A	ss715646896	1224240	2040423
BARCPV_1.0_Chr04_1270861_A_G	ss715646898	1270861	1381835
BARCPV_1.0_Chr04_1278699_C_T	ss715646899	1278699	1389673
BARCPV_1.0_Chr04_1386459_A_G	ss715639413	1386459	1496611
BARCPV_1.0_Chr04_1433996_G_A	ss715646903	1433996	1543024
BARCPV_1.0_Chr04_1497419_A_C	ss715646909	1497419	1604207
BARCPV_1.0_Chr04_1503482_T_C	ss715646910	1503482	1610074
BARCPV_1.0_Chr04_1575721_A_G	ss715649973	1575721	1679018

BARCPV_1.0_Chr04_1627690_A_G	ss715649971	1627690	1738973
BARCPV_1.0_Chr04_1721502_G_A	ss715647820	1721502	1834806
BARCPV_1.0_Chr04_1728453_G_T	ss715647821	1728453	1842115
BARCPV_1.0_Chr04_1739357_G_A	ss715647822	1739357	1853021
BARCPV_1.0_Chr04_1745263_T_G	ss715647823	1745263	1858930
BARCPV_1.0_Chr04_1827663_C_T	ss715647806	1827663	2110777
BARCPV_1.0_Chr04_1833878_A_G	ss715647807	1833878	2116992
BARCPV_1.0_Chr04_1845589_T_C	ss715647808	1845589	2131629
BARCPV_1.0_Chr04_1865273_G_T	ss715647811	1865273	2151313
BARCPV_1.0_Chr04_1889393_C_T	ss715647813	1889393	2175434
BARCPV_1.0_Chr04_1982297_C_T	ss715647819	1982297	2274277
BARCPV_1.0_Chr04_2000276_A_G	ss715650013	2000276	2292256
BARCPV_1.0_Chr04_2007584_T_C	ss715650008	2007584	2299564
BARCPV_1.0_Chr04_2013445_C_T	ss715650009	2013445	2305425
BARCPV_1.0_Chr04_2026101_T_C	ss715650010	2026101	#N/A
BARCPV_1.0_Chr04_2059126_T_C	ss715650014	2059126	#N/A
BARCPV_1.0_Chr04_2064654_G_A	ss715650015	2064654	2356621
BARCPV_1.0_Chr04_2072060_T_C	ss715650016	2072060	2364027
BARCPV_1.0_Chr04_2136250_A_G	ss715646249	2136250	2410894
BARCPV_1.0_Chr04_2142286_C_A	ss715646248	2142286	2416930
BARCPV_1.0_Chr04_2153405_A_G	ss715646246	2153405	2428047
BARCPV_1.0_Chr04_2276976_T_C	ss715646239	2276976	2553713
BARCPV_1.0_Chr04_2608903_A_G	ss715646218	2608903	2893246
BARCPV_1.0_Chr04_2670845_A_C	ss715646215	2670845	2945885
BARCPV_1.0_Chr04_2676413_C_T	ss715646214	2676413	#N/A
BARCPV_1.0_Chr04_2758051_G_A	ss715646204	2758051	3035540
BARCPV_1.0_Chr04_2780607_C_T	ss715646200	2780607	3058095
BARCPV_1.0_Chr04_2854887_T_C	ss715646233	2854887	3131385
BARCPV_1.0_Chr04_2862612_G_T	ss715646228	2862612	3139176
BARCPV_1.0_Chr04_2880210_A_G	ss715646217	2880210	3156782
BARCPV_1.0_Chr04_2886459_T_C	ss715646213	2886459	#N/A
BARCPV_1.0_Chr04_2894249_G_T	ss715646201	2894249	3170822
BARCPV_1.0_Chr04_2903307_C_T	ss715646224	2903307	3179880
BARCPV_1.0_Chr04_2966427_A_G	ss715649002	2966427	3244521
BARCPV_1.0_Chr04_2981879_A_G	ss715649001	2981879	3259973
BARCPV_1.0_Chr04_3006244_C_T	ss715649010	3006244	3283618
BARCPV_1.0_Chr04_3018176_G_A	ss715649009	3018176	3295550
BARCPV_1.0_Chr04_3027795_C_T	ss715649008	3027795	3305169
BARCPV_1.0_Chr04_3041884_T_C	ss715649007	3041884	3319376
BARCPV_1.0_Chr04_3054966_A_G	ss715649006	3054966	3332458
BARCPV_1.0_Chr04_3085453_T_C	ss715647287	3085453	3394426
BARCPV_1.0_Chr04_3135346_C_A	ss715647293	3135346	3444416
BARCPV_1.0_Chr04_3143476_A_C	ss715647294	3143476	3452546
BARCPV_1.0_Chr04_3170316_A_G	ss715647284	3170316	3479386

BARCPV_1.0_Chr04_3176573_G_A	ss715647285	3176573	3486066
BARCPV_1.0_Chr04_3199273_C_A	ss715647286	3199273	3508766
BARCPV_1.0_Chr04_3254820_G_A	ss715650368	3254820	3563198
BARCPV_1.0_Chr04_3279111_G_A	ss715650366	3279111	3587489
BARCPV_1.0_Chr04_3315474_T_C	ss715650365	3315474	3594648
BARCPV_1.0_Chr04_3331454_G_A	ss715650370	3331454	#N/A
BARCPV_1.0_Chr04_3492292_A_G	ss715646816	3492292	3759797
BARCPV_1.0_Chr04_3523277_T_G	ss715646812	3523277	3792798
BARCPV_1.0_Chr04_3532372_G_T	ss715646811	3532372	3802863
BARCPV_1.0_Chr04_3544877_G_A	ss715646810	3544877	3815368
BARCPV_1.0_Chr04_3575468_T_C	ss715646807	3575468	3845959
BARCPV_1.0_Chr04_3715072_C_T	ss715649005	3715072	3341703
BARCPV_1.0_Chr04_3768718_C_T	ss715646796	3768718	3972650
BARCPV_1.0_Chr04_3778168_C_T	ss715646795	3778168	3982098
BARCPV_1.0_Chr04_3797196_A_G	ss715646793	3797196	4001126
BARCPV_1.0_Chr04_3830179_G_A	ss715646792	3830179	4034109
BARCPV_1.0_Chr04_3840559_G_A	ss715646790	3840559	4044489
BARCPV_1.0_Chr04_3847808_C_T	ss715646788	3847808	4056126
BARCPV_1.0_Chr04_3905677_A_C	ss715646784	3905677	4114001
BARCPV_1.0_Chr04_3937414_T_C	ss715646782	3937414	#N/A
BARCPV_1.0_Chr04_4181353_C_A	ss715648122	4181353	4411280
BARCPV_1.0_Chr04_4192584_T_C	ss715648121	4192584	4422511
BARCPV_1.0_Chr04_4260101_A_G	ss715648117	4260101	4489815
BARCPV_1.0_Chr04_4274980_A_C	ss715648116	4274980	4504538
BARCPV_1.0_Chr04_4286405_T_C	ss715648115	4286405	4515963
BARCPV_1.0_Chr04_4303204_A_G	ss715648114	4303204	4532562
BARCPV_1.0_Chr04_4308684_A_G	ss715639785	4308684	4537962
BARCPV_1.0_Chr04_4342456_T_C	ss715648126	4342456	4572255
BARCPV_1.0_Chr04_4410410_A_C	ss715649532	4410410	4639284
BARCPV_1.0_Chr04_4429048_G_T	ss715640556	4429048	4664654
BARCPV_1.0_Chr04_4489669_A_C	ss715649533	4489669	4731594
BARCPV_1.0_Chr04_4497307_T_C	ss715649528	4497307	4739232
BARCPV_1.0_Chr04_4509382_C_T	ss715640555	4509382	4751308
BARCPV_1.0_Chr04_4563346_C_T	ss715643858	4563346	4810753
BARCPV_1.0_Chr04_4629011_A_G	ss715647594	4629011	4884604
BARCPV_1.0_Chr04_4671016_A_G	ss715647600	4671016	4928498
BARCPV_1.0_Chr04_4679535_C_T	ss715647601	4679535	4937017
BARCPV_1.0_Chr04_4717287_G_A	ss715647590	4717287	4974771
BARCPV_1.0_Chr04_4730031_A_G	ss715647591	4730031	4987517
BARCPV_1.0_Chr04_4753192_G_A	ss715647592	4753192	5010646
BARCPV_1.0_Chr04_4988501_G_A	ss715639594	4988501	5241533
BARCPV_1.0_Chr04_5083634_A_G	ss715648332	5083634	5335714
BARCPV_1.0_Chr04_5109467_C_A	ss715648318	5109467	5361550
BARCPV_1.0_Chr04_5160162_T_C	ss715648320	5160162	5412867

BARCPV_1.0_Chr04_5236970_T_C	ss715648327	5236970	5489705
BARCPV_1.0_Chr04_5246330_C_T	ss715648328	5246330	5499065
BARCPV_1.0_Chr04_5489750_G_A	ss715649296	5489750	5744865
BARCPV_1.0_Chr04_5625817_A_C	ss715648226	5625817	5880971
BARCPV_1.0_Chr04_5657565_C_T	ss715648228	5657565	5913827
BARCPV_1.0_Chr04_5700111_T_C	ss715648222	5700111	5956690
BARCPV_1.0_Chr04_5975931_G_T	ss715641933	5975931	6273267
BARCPV_1.0_Chr04_5978333_G_A	ss715641932	5978333	6275669
BARCPV_1.0_Chr04_5995544_T_C	ss715641934	5995544	6296686
BARCPV_1.0_Chr04_6157894_T_C	ss715640044	6157894	6489841
BARCPV_1.0_Chr04_6241077_G_A	ss715640046	6241077	6576995
BARCPV_1.0_Chr04_6493282_T_G	ss715641595	6493282	6847471
BARCPV_1.0_Chr04_6540165_C_T	ss715644605	6540165	6893920
BARCPV_1.0_Chr04_6915759_G_A	ss715644065	6915759	7170431
BARCPV_1.0_Chr04_7188634_A_G	ss715649153	7188634	7452824
BARCPV_1.0_Chr04_7366314_C_T	ss715650214	7366314	7629662
BARCPV_1.0_Chr04_7392047_C_T	ss715650213	7392047	7655766
BARCPV_1.0_Chr04_7708046_C_T	ss715639841	7708046	8026040
BARCPV_1.0_Chr04_7752685_A_C	ss715639839	7752685	8070843
BARCPV_1.0_Chr04_8543342_G_A	ss715639598	8543342	8888286
BARCPV_1.0_Chr04_8941660_A_G	ss715639283	8941660	9301023
BARCPV_1.0_Chr04_9107757_G_A	ss715639280	9107757	9461806
BARCPV_1.0_Chr04_9259094_A_C	ss715646644	9259094	9651954
