# Supplementary material for: Fine Mapping of the Co-12 Anthracnose Resistance Gene in the Andean Common Bean Cultivar in Brazil
Source: Plants (Basel). 2026 Mar 18;15(6):931. doi: 10.3390/plants15060931 (PMC13030795; doi:10.3390/plants15060931)
Supplement: Supplementary file 1 [file plants-15-00931-s001.zip › plants-4177286-Table S4.pdf]

Table S4: Genotyping of 172 $F_2$ plants from the Jalo Vermelho × Crioulo 159 cross with five SSR markers and inoculated with race 1545 of *C. lindemuthianum* .

| Position on Pv04 (bp) (v2.1) | | 94873 | 136199 | 269985 | 430389 | 2145580 |
|---|---|---|---|---|---|---|
| $F_2$ | Reaction of $F_{2:3}$ families to *C. lindemuthianum* race 1545 | SSR 4557 | SSR 4570 | SSR 4574 | SSR 4577 | SSR 4619 |
| Jalo Vermelho | Resistant | BB | BB | BB | BB | BB |
| Crioulo 159 | Susceptible | AA | AA | AA | AA | AA |
| 1 | Resistant | BB | BB | BB | BB | BB |
| 2 | Heterozygous | AB | AB | AB | AB | AB |
| 3 | Heterozygous | AB | AB | AB | AB | AB |
| 4 | Resistant | BB | BB | BB | BB | BB |
| 5 | Heterozygous | AB | AB | AB | AB | AB |
| 6 | Heterozygous | AB | AB | AB | AB | AB |
| 7 | Resistant | BB | BB | BB | BB | BB |
| 8 | Heterozygous | AB | AB | AB | AB | AB |
| 9 | Heterozygous | AB | AB | AB | AB | AB |
| 10 | Heterozygous | AB | AB | AB | AB | AB |
| 11 | Susceptible | AA | AA | AA | AA | AA |
| 12 | Heterozygous | AB | AB | AB | AB | AB |
| 13 | Susceptible | AA | AA | AA | AA | AA |
| 14 | Heterozygous | AB | AB | AB | AB | AB |
| 15 | Resistant | BB | BB | BB | BB | BB |
| 16 | Heterozygous | AB | AB | AB | AB | AB |
| 17 | Resistant | BB | BB | BB | BB | BB |
| 18 | Heterozygous | AB | AB | AB | AB | AB |
| 19 | Resistant | BB | BB | BB | BB | BB |
| 20 | Heterozygous | AB | AB | AB | AB | AB |
| 21 | Susceptible | AA | AA | AA | AA | AA |
| 22 | Resistant | BB | BB | BB | BB | BB |
| 23 | Resistant | BB | BB | BB | BB | BB |
| 24 | Heterozygous | AB | AB | AB | AB | AB |
| 25 | Susceptible | AA | AA | AA | AA | AA |
| 26 | Resistant | BB | BB | BB | BB | BB |
| 27 | Heterozygous | AB | AB | AB | AB | AB |
| 28 | Heterozygous | AB | AB | AB | AB | AB |
| 29 | Susceptible | AA | AA | AA | AA | AA |
| 30 | Heterozygous | AB | AB | AB | AB | AB |
| 31 | Resistant | BB | BB | BB | BB | BB |
| 32 | Resistant | BB | BB | BB | BB | BB |
| 33 | Heterozygous | AB | AB | AB | AB | AB |
| 34 | Susceptible | AA | AA | AA | AA | AA |
| 35 | Susceptible | AA | AA | AA | AA | AA |
| 36 | Heterozygous | AB | AB | AB | AB | AB |
| 37 | Resistant | BB | BB | BB | BB | BB |
| 38 | Susceptible | AA | AA | AA | AA | AA |
| 39 | Heterozygous | AB | AB | AB | AB | AB |
| 40 | Heterozygous | AB | AB | AB | AB | AB |
| 41 | Heterozygous | AB | AB | AB | AB | AB |
| 42 | Susceptible | AA | AA | AA | AA | AA |
| 43 | Susceptible | AA | AA | AA | AA | AA |
| 44 | Resistant | BB | BB | BB | BB | BB |
| 45 | Heterozygous | AB | AB | AB | AB | AB |
| 46 | Heterozygous | AB | AB | AB | AB | AB |
| 47 | Resistant | BB | BB | BB | BB | BB |
| 48 | Susceptible | AA | AA | AA | AA | AA |
| 49 | Heterozygous | AB | AB | AB | AB | AB |
| 50 | Resistant | BB | BB | BB | BB | BB |
| 51 | Heterozygous | AB | AB | AB | AB | AB |
| 52 | Resistant | BB | BB | BB | BB | BB |
| 53 | Heterozygous | AB | AB | AB | AB | AB |
| 54 | Susceptible | AA | AA | AA | AA | AA |

| | | | | | | |
|---|---|---|---|---|---|---|
| 55 | Heterozygous | AB | AB | AB | AB | AB |
| 56 | Heterozygous | AB | AB | AB | AB | AB |
| 57 | Heterozygous | AB | AB | AB | AB | AB |
| 58 | Resistant | BB | BB | BB | BB | BB |
| 59 | Susceptible | AA | AA | AA | AA | AA |
| 60 | Susceptible | AA | AA | AA | AA | AA |
| 61 | Susceptible | AA | AA | AA | AA | AA |
| 62 | Heterozygous | AB | AB | AB | AB | AB |
| 63 | Resistant | BB | BB | BB | BB | BB |
| 64 | Heterozygous | AB | AB | AB | AB | AB |
| 65 | Heterozygous | AB | AB | AB | AB | AB |
| 66 | Resistant | BB | BB | BB | BB | BB |
| 67 | Heterozygous | AB | AB | AB | AB | AB |
| 68 | Susceptible | AA | AA | AA | AA | AA |
| 69 | Heterozygous | AB | AB | AB | AB | AB |
| 70 | Heterozygous | AB | AB | AB | AB | AB |
| 71 | Susceptible | AA | AA | AA | AA | AA |
| 72 | Susceptible | AA | AA | AA | AA | AA |
| 73 | Susceptible | AA | AA | AA | AA | AA |
| 74 | Resistant | BB | BB | BB | BB | BB |
| 75 | Heterozygous | AB | AB | AB | AB | AB |
| 76 | Susceptible | AA | AA | AA | AA | AA |
| 77 | Susceptible | AA | AA | AA | AA | AA |
| 78 | Heterozygous | AB | AB | AB | AB | AB |
| 79 | Resistant | BB | BB | BB | BB | BB |
| 80 | Resistant | BB | BB | BB | BB | BB |
| 81 | Resistant | BB | BB | BB | BB | BB |
| 82 | Heterozygous | AB | AB | AB | AB | AB |
| 83 | Heterozygous | AB | AB | AB | AB | AB |
| 84 | Heterozygous | AB | AB | AB | AB | AB |
| 85 | Heterozygous | AB | AB | AB | AB | AB |
| 86 | Heterozygous | AB | AB | AB | AB | AB |
| 87 | Heterozygous | AB | AB | AB | AB | AB |
| 88 | Heterozygous | AB | AB | AB | AB | AB |
| 89 | Susceptible | AA | AA | AA | AA | AA |
| 90 | Heterozygous | AB | AB | AB | AB | AB |
| 91 | Heterozygous | AB | AB | AB | AB | AB |
| 92 | Susceptible | AA | AA | AA | AA | AA |
| 93 | Heterozygous | AB | AB | AB | AB | AB |
| 94 | Heterozygous | AB | AB | AB | AB | AB |
| 95 | Susceptible | AA | AA | AA | AA | AA |
| 96 | Susceptible | AA | AA | AA | AA | AA |
| 97 | Heterozygous | AB | AB | AB | AB | AB |
| 98 | Heterozygous | AB | AB | AB | AB | AB |
| 99 | Heterozygous | AB | AB | AB | AB | AB |
| 100 | Heterozygous | AB | AB | AB | AB | AB |
| 101 | Susceptible | AA | AA | AA | AA | AA |
| 102 | Resistant | BB | BB | BB | BB | BB |
| 103 | Susceptible | AA | AA | AA | AA | AA |
| 104 | Resistant | BB | BB | BB | BB | BB |
| 105 | Heterozygous | AB | AB | AB | AB | AB |
| 106 | Heterozygous | AB | AB | AB | AB | AB |
| 107 | Heterozygous | AB | AB | AB | AB | AB |
| 108 | Resistant | BB | BB | BB | BB | BB |
| 109 | Susceptible | AA | AA | AA | AA | AA |
| 110 | Susceptible | AA | AA | AA | AA | AA |
| 111 | Heterozygous | AB | AB | AA | AA | AA |
| 112 | Susceptible | AA | AA | AA | AA | AA |
| 113 | Susceptible | AA | AB | AB | AB | AB |
| 114 | Heterozygous | AB | AB | AB | AB | AB |
| 115 | Heterozygous | AB | AB | AB | AB | AB |
| 116 | Resistant | BB | BB | BB | BB | BB |
| 117 | Heterozygous | AB | AB | AB | AB | AB |

| | | | | | | |
|---|---|---|---|---|---|---|
| 118 | Resistant | BB | BB | BB | BB | BB |
| 119 | Heterozygous | AB | AB | AB | AB | AB |
| 120 | Resistant | BB | BB | BB | BB | BB |
| 121 | Resistant | BB | BB | BB | BB | BB |
| 122 | Resistant | BB | BB | BB | BB | BB |
| 123 | Heterozygous | AB | AB | AB | AB | AB |
| 124 | Resistant | BB | BB | BB | BB | BB |
| 125 | Resistant | BB | BB | BB | BB | BB |
| 126 | Resistant | BB | BB | BB | BB | BB |
| 127 | Heterozygous | AB | AB | AB | AB | AB |
| 128 | Heterozygous | AB | AB | AB | AB | AB |
| 129 | Heterozygous | AB | AB | AB | AB | AB |
| 130 | Heterozygous | AB | AB | AB | AB | AB |
| 131 | Heterozygous | AB | AB | AB | AB | AB |
| 132 | Heterozygous | AB | AB | AB | AB | AB |
| 133 | Resistant | BB | BB | BB | BB | BB |
| 134 | Heterozygous | AB | AB | AB | AB | AB |
| 135 | Resistant | BB | BB | BB | BB | BB |
| 136 | Resistant | BB | BB | BB | BB | BB |
| 137 | Resistant | BB | BB | BB | BB | BB |
| 138 | Susceptible | AA | AA | AA | AA | AA |
| 139 | Heterozygous | AB | AB | AB | AB | AB |
| 140 | Heterozygous | AB | AB | AB | AB | AB |
| 141 | Heterozygous | AB | AB | AB | AB | AB |
| 142 | Heterozygous | AB | AB | AB | AB | AB |
| 143 | Heterozygous | AB | AB | AB | AB | AB |
| 144 | Heterozygous | AB | AB | AB | AB | AB |
| 145 | Susceptible | AA | AA | AA | AA | AA |
| 146 | Susceptible | AA | AA | AA | AA | AA |
| 147 | Resistant | BB | BB | BB | BB | BB |
| 148 | Resistant | BB | BB | BB | BB | BB |
| 149 | Heterozygous | AB | AB | AB | AB | AB |
| 150 | Resistant | BB | BB | BB | BB | BB |
| 151 | Susceptible | AA | AA | AA | AA | AA |
| 152 | Heterozygous | AB | AB | AB | AB | AB |
| 153 | Resistant | BB | BB | BB | BB | BB |
| 154 | Heterozygous | AB | AB | AB | AB | AB |
| 155 | Resistant | BB | BB | BB | BB | BB |
| 156 | Susceptible | AA | AA | AA | AA | AA |
| 157 | Susceptible | AA | AA | AA | AA | AA |
| 158 | Susceptible | AA | AA | AA | AA | AA |
| 159 | Resistant | BB | BB | BB | BB | BB |
| 160 | Resistant | BB | BB | BB | BB | BB |
| 161 | Heterozygous | AB | AB | AB | AB | AB |
| 162 | Heterozygous | AB | AB | AB | AB | AB |
| 163 | Heterozygous | AB | AB | AB | AB | AB |
| 164 | Heterozygous | AB | AB | AB | AB | AB |
| 165 | Heterozygous | AB | AB | AB | AB | AB |
| 166 | Heterozygous | AB | AB | AB | AB | AB |
| 167 | Susceptible | AA | AA | AA | AA | AA |
| 168 | Susceptible | AA | AA | AA | AA | AA |
| 169 | Heterozygous | AB | AA | AA | AA | AA |
| 170 | Susceptible | AA | AA | AA | AA | AA |
| 171 | Heterozygous | AB | AA | AA | AA | AA |
| 172 | Susceptible | AA | AA | AA | AA | AA |

BB = resistant; AA = susceptible; AB = heterozygous; -- = missing data

Evaluation was conducted using the severity scale by van Schoonhoven and Pastor-Corrales (1987).
