# Supplementary material for: Fine Mapping of the Co-12 Anthracnose Resistance Gene in the Andean Common Bean Cultivar in Brazil
Source: Plants (Basel). 2026 Mar 18;15(6):931. doi: 10.3390/plants15060931 (PMC13030795; doi:10.3390/plants15060931)
Supplement: Supplementary file 1 [file plants-15-00931-s001.zip › plants-4177286-Table S5.pdf]

Table S5. Reactions of common bean cultivars inoculated with Andean and Mesoamerican races of *Colletotrichum lindemuthianum* . Adapted from [49].

Cultivars	Gene pool	Genes	Races of *Colletotrichum lindemuthianum*												
			**2**	**7**	**19**	**23**	**39**	**55**	9	65	73	89	449	453	1545
Michelite	M	*Co-11*	R	S[d]	S	S	S	S	S	S	S	S	S	S	S
**MDRK**	A	*Co-1*	S	S	S	S	S	S	R	R	R	R	R	R	R
**Perry Marrow**	A	*Co-1*^{3}	R	S	R	S	S	S	R	R	R	R	R	S	R
Cornell 49-242	M	*Co-2*	R	R	S	R	R	R	S	R	S	S	R	R	S
**Widusa**	A	*Co-1*^{5}	R	R	R	S	R	S	R	R	R	S	R	R	R
**Kaboon**	A	*Co-1*^{2}	R	R	R	R	S	S	R	R	R	R	R	R	R
Mexico 222	M	*Co-3*	R	R	R	R	R	R	R	S	S	S	S	S	R
PI 207262	M	*Co-4*^{3} /*Co-3*^{3}	R	R	R	R	R	R	R	R	R	R	S	S	R
TO	M	*Co-4*	R	R	R	R	R	R	R	R	R	R	S	S	R
TU	M	*Co-5*	R	R	R	R	R	R	R	R	R	R	R	R	S
AB136	M	*Co-6/co-8*	R	R	R	R	R	R	R	R	R	R	R	R	S
G2333	M	*Co-4*^{2} /*Co-5*^{2} /*Co-3*^{5}	R	R	R	R	R	R	R	R	R	R	R	R	R
**Jalo Vermelho**	A	*Co-12*	R	S	S	R	S	R	R	R	S	R	R	R	R
**Crioulo 159**	M	*Co-16*	R	NE	R	R	R	R	NE	S	R	NE	R	S	S
**Paloma**	A	*CoPv01*^{PA}	S	S	S	R	R	R	S	R	R	S	S	S	R
**AND 277**	A	*Co-1*^{4}	R	S	S	R	S	R	S	R	R	R	R	R	R
**Jalo Listras Pretas**	A	*Co-13*	S	S	S	S	S	S	R	R	R	R	S	S	R
**Pitanga**	A	*Co-14*	R	S	R	R	S	R	S	R	R	R	S	S	S
**Corinthiano**	A	*Co-15*	R	S	S	R	NE	S	S	R	R	R	S	S	R
**Amendoim Cavalo**	A	*CoPv01*^{AC}	R	R	R	R	R	R	R	R	R	R	S	S	R
**Beija Flor**	A	*Co-Bf*	NE	NE	R	R	S	S	NE	R	R	NE	R	R	R
**Jalo EEP 558**	A	*Co-w/Co-x/Co-y/Co-z*	S	S	S	NE	NE	S	NE	R	R	S	R	R	R

Cultivars and races of *C. lindemuthianum* highlighted (bolded) are Andean; others are Mesoamerican. M = Mesoamerican; A = Andean; R = Resistant; S = Susceptible; NE= Not evaluated.
